# ResMAP—a saturation mutagenesis platform enabling parallel profiling of target-specific resistance-conferring mutations in *Plasmodium*

**DOI:** 10.1128/mbio.01708-24

**Published:** 2024-08-27

**Authors:** Richard J. Wall, Stuart A. MacGowan, Irene Hallyburton, Aisha J. Syed, Sowmya Ajay Castro, Gourav Dey, Rachel Milne, Stephen Patterson, Jody Phelan, Natalie Wiedemar, Susan Wyllie

**Affiliations:** 1 Wellcome Center for Anti-infectives Research, Division of Biological Chemistry and Drug Discovery, School of Life Sciences, University of Dundee, Dow Street, Dundee, United Kingdom; 2 Division of Computational Biology, School of Life Sciences, University of Dundee, Dundee, United Kingdom; 3 Drug Discovery Unit, Wellcome Center for Anti-infectives Research, Division of Biological Chemistry and Drug Discovery, University of Dundee, Dundee, United Kingdom; 4 Division of Molecular Microbiology, School of Life Sciences, University of Dundee, Dundee, United Kingdom; 5 Department of Infection Biology, Faculty of Infectious and Tropical Diseases, London School of Hygiene and Tropical Medicine, London, United Kingdom; Rutgers-New Jersey Medical School, Newark, New Jersey, USA

**Keywords:** mutagenesis, *Plasmodium*, drug targets, drug-resistance mechanisms, malaria, antimalarial agents

## Abstract

New and improved drugs are required for the treatment and ultimate eradication of malaria. The efficacy of front-line therapies is now threatened by emerging drug resistance; thus, new tools to support the development of drugs with a lower propensity for resistance are needed. Here, we describe the development of a RESistance Mapping And Profiling (ResMAP) platform for the identification of resistance-conferring mutations in *Plasmodium* drug targets. Proof-of-concept studies focused on interrogating the antimalarial drug target, *Plasmodium falciparum* lysyl tRNA synthetase (*Pf*KRS). Saturation mutagenesis was used to construct a plasmid library encoding all conceivable mutations within a 20-residue span at the base of the *Pf*KRS ATP-binding pocket. The superior transfection efficiency of *Plasmodium knowlesi* was exploited to generate a high coverage parasite library expressing *Pf*KRS bearing all possible amino acid changes within this region of the enzyme. The selection of the library with *Pf*KRS inhibitors, cladosporin and DDD01510706, successfully identified multiple resistance-conferring substitutions. Genetic validation of a subset of these mutations confirmed their direct role in resistance, with computational modeling used to dissect the structural basis of resistance. The application of ResMAP to inform the development of resistance-resilient antimalarials of the future is discussed.

**IMPORTANCE:**

An increase in treatment failures for malaria highlights an urgent need for new tools to understand and minimize the spread of drug resistance. We describe the development of a RESistance Mapping And Profiling (ResMAP) platform for the identification of resistance-conferring mutations in *Plasmodium* spp, the causative agent of malaria. Saturation mutagenesis was used to generate a mutation library containing all conceivable mutations for a region of the antimalarial-binding site of a promising drug target, *Plasmodium falciparum* lysyl tRNA synthetase (*Pf*KRS). Screening of this high-coverage library with characterized *Pf*KRS inhibitors revealed multiple resistance-conferring substitutions including several clinically relevant mutations. Genetic validation of these mutations confirmed resistance of up to 100-fold and computational modeling dissected their role in drug resistance. We discuss potential applications of this data including the potential to design compounds that can bypass the most serious resistance mutations and future resistance surveillance.

## INTRODUCTION

In 2022 alone, malaria was responsible for approximately 608,000 deaths, primarily African children under 5 (1). After several years of decreasing mortality rates, progress in controlling malaria has now stalled and, in some areas, reversed (1). The disease is caused by infection with *Plasmodium spp*., with *Plasmodium falciparum* and *Plasmodium vivax* responsible for the majority of severe cases and deaths. Despite a relatively healthy arsenal of antimalarial therapies, drug resistance is a constant threat, so there is a pressing need for new drugs to provide chemoprotection, prevent transmission, and treat relapsing malaria. Alongside new drug therapies, novel strategies and tools to minimize the threat of drug resistance are essential.

In recent years, there has been a decided change of emphasis from antimalarial drug discovery driven through purely phenotypic routes to target-focused and structure-enabled drug discovery (2). A major advantage of the target-focused approach is the fact that the compound-binding site is well characterized, allowing active compounds to be optimized to improve affinity for the target and enhance selectivity over human homologues. However, it could be argued that these single-target compounds are more vulnerable to the emergence of drug resistance specifically through target mutation. Identifying resistance-conferring mutations associated with a specific compound-target pair commonly involves time-consuming *in vitro* resistance selections, followed by whole-genome sequencing. Often, this approach fails to identify the full range of resistance-conferring single nucleotide polymorphisms (SNPs) in one go, and multiple selections are required to reveal the true resistance landscape. As an example, consider cipargamin, a promising antimalarial drug currently in stage II clinical trials and known to target P-type cation ATPase (*Pf*ATP4). To date, 37 different mutations conferring resistance to ATP4-targeting compounds have been identified from a myriad of *in vitro* resistance selections (3); this list is very likely non-exhaustive. Recently, one of these mutations (G358S) was rapidly selected during human trials of cipargamin, highlighting the relevance of these mutations once drugs are in clinical use (4). Although G358S had been associated with resistance to other ATP4-targeting compounds, its role in cipargamin resistance was only recognized after this trial (3, 5). The fact that this mutation, which results in a >4,000-fold reduction in drug susceptibility, had not been directly associated with cipargamin prior to clinical trials is a major concern (5). The ability to compile a comprehensive list of all conceivable mutations capable of conferring resistance to a new antimalarial would be highly advantageous, providing a better understanding of the resistance potential associated with specific drug targets. Such data could also be compared to existing field isolate data to confirm whether there may be *Plasmodium* strains that are naturally resistant to drugs in development.

A comprehensive understanding of the resistance landscape of a particular molecular target could also be exploitable by medicinal chemists to develop compounds with comparatively lower resistance potential. In combination with structural modeling, knowledge of resistance-conferring mutations and the levels of resistance achievable through mutation of specific amino acids in the compound binding pocket could be used to guide the development of compounds capable of bypassing the highest levels of resistance (6). Thus, an ability to rapidly and comprehensively identify all conceivable resistance-associated mutations could support drug discovery efforts and assist in monitoring the emergence of drug resistance in the clinic.

Approaches to identify all target-associated resistance-conferring mutations in *Plasmodium* have not been reported previously. Saturation mutagenesis has been used to investigate the functional effects of SNPs on a key cancer gene (7) and to probe the effect of mutations on the interaction between the SARS-CoV-2 spike and host ACE2 proteins (8, 9). Other gene-wide mutagenesis studies include the comprehensive characterization of PncA polymorphisms that confer resistance to the anti-tubercular drug pyrazinamide (10), functional dissection of the bacterial ABC transporter EfrCD (11), and the guiding of antibiotic design targeting three key *Escherichia coli* drug targets (12).

Here, we describe the development of a RESistance Mapping And Profiling (ResMAP) platform, a target-specific mutation library engineered in *Plasmodium knowlesi* to probe the immediate binding context of inhibitors targeting the ATP-binding pocket of *P. falciparum* lysyl-tRNA synthetase (*Pf*KRS) (13, 14). The library comprises subpopulations of parasites each expressing a *Pf*KRS bearing a single amino acid substitution in the inhibitor-binding pocket. Every conceivable amino acid substitution within this region, spanning a total of 400 amino acids in total, is represented within the library. The selection of the library with established KRS inhibitors cladosporin (14) and DDD01510706 (13) led to the identification of multiple amino acid changes capable of driving resistance to these compounds. Several resistance-conferring mutations not previously revealed through conventional *in vitro* selections were identified, many providing significant levels of resistance (>100-fold) to cladosporin and DDD01510706. These studies provide proof-of-concept for the generation of saturation mutagenesis libraries in *Plasmodium* to support the development of new antimalarials with reduced resistance potential. Strategies to evolve and improve the library and potential uses for the resulting data sets are discussed.

## RESULTS

### Expression of *P. falciparum* lysyl-tRNA synthetase in *P. knowlesi*

**Fig 1:**
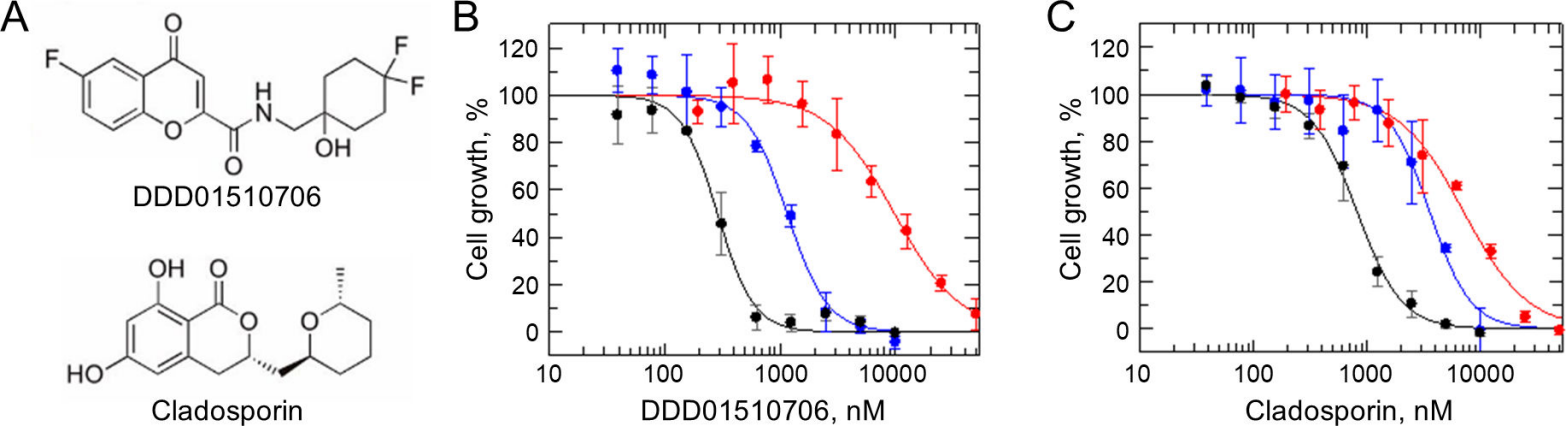
*Pf*KRS expression in *P. knowlesi*. (**A**) Chemical structures of DDD01510706 and cladosporin. (**B**) Dose-response curves of DDD01510706 against *P. knowlesi* wild-type (WT; black), *Pf*KRS^WT^ (blue), and *Pf*KRS^S344L^ (red) expressing parasites. EC_50_ values were WT: 286 ± 16 nM, *Pf*KRS^WT^: 1150 ± 83 nM and *Pf*KRS^S344L^: 9820 ± 760 nM. (**C**) Dose-response curves of cladosporin against *P. knowlesi* WT (black), *Pf*KRS^WT^ (blue) and *Pf*KRS^S344L^ (red) expressing parasites. EC_50_ values were WT: 823 ± 35, *Pf*KRS^WT^: 3,580 ± 296 nM, and *Pf*KRS^S344L^: 7,110 ± 580 nM. Representative dose-response curves consisting of three technical replicates ±SD shown.

To provide proof-of-concept for our deep mutagenesis screening platform (ResMAP), we focused on investigating mutations capable of conferring resistance to inhibitors targeting *Pf*KRS (also referred to as KRS1). This aminoacyl-tRNA synthetase was considered a particularly attractive target since a high-resolution crystal structure and well-defined, highly selective inhibitors (DDD01510706 and cladosporin) are available (13–15) (Fig. 1A). To comprehensively profile mutations capable of conferring resistance to specific *P. falciparum* KRS inhibitors, a library with all conceivable mutations within part of the inhibitor binding site was generated.

Poor transfection efficiency precluded the construction of a library of this complexity in *P. falciparum*. As an alternative, we took advantage of the superior transfection efficiency of *P. knowlesi* (16). This human-adapted *Plasmodium* parasite is often used as a surrogate for *P. falciparum* since it can readily express proteins from multiple other *Plasmodium* species (17–19). In this particular case, *P. falciparum* (PF3D7_135010) and *P. knowlesi* (PKNH_1250800) KRS share relatively high-sequence homology (81%; Fig. S1).

All *Pf*KRS inhibitors reported to date bind competitively to the ATP-binding pocket of the enzyme. Since *Plasmodium* is a haploid parasite and KRS is an essential enzyme, many mutations are likely to be detrimental for enzyme function, and parasites expressing these mutants would immediately be lost from this library. Thus, to guarantee that all conceivable mutations are represented in our screens, ensuring that residues that cannot tolerate mutation as well as those that can are identified, we opted to introduce an ectopic copy of *Pf*KRS rather than interfere with the endogenous *P. knowlesi* gene. Previous studies demonstrate that episomal copy number in *Plasmodium* self-regulates, and high copy number is only achievable under selective pressure (20). In this instance, regulation will occur under pyrimethamine (PYR) and KRS inhibitor selection and will be concentration dependent. Our previous studies have shown that the overexpression of the *P. falciparum* protein (*Pf*KRS^WT^) in *P. falciparum* confers resistance to compounds specifically targeting *Pf*KRS (15). Importantly, we confirmed that the overexpression of *Pk*KRS in *P. knowlesi* had a similar impact on KRS inhibitor susceptibility (Table 1; Fig. S2). In the current study, we investigated the impact of *Pf*KRS^WT^ expression in *P. knowlesi* via transfection with an adapted pkconGFP episomal expression vector with *Pf*KRS under the control of a *Pk*HSP70 promotor (16). Using label-free proteomics, we confirmed that *Pf*KRS^WT^ was expressed in *P. knowlesi* but were unable to accurately quantify the precise levels of *Pf*KRS expression relative to endogenous *Pk*KRS due to high-sequence similarity. Furthermore, quantification is also limited since copy number levels will be governed not only by pyrimethamine but also by the presence and concentration of KRS inhibitor (Fig. S3). Ectopic expression of the *Pf*KRS^WT^ in *P. knowlesi* led to a four- and eightfold reproducible shift in susceptibility to DDD01510706 and cladosporin, respectively (Fig. 1B and C; Table 1).

**TABLE 1:** EC_50_ values for WT and transgenic *P. knowlesi* cell lines—weighted mean ± SD of ≥2 independent experiments each comprising three technical replicates^*a*^

Cell line	EC_50_ values, nM	
	DDD01510706	Cladosporin
WT	211 ± 7.6	493 ± 29
*Pf*KRS^WT^	828 ± 68 (4)	4,030 ± 355 (8)
*Pf*KRS^S344L^	10,600 ± 755 (50)	6,510 ± 439 (13)
*Pf*KRS^V328S^	10,400 ± 1,060 (50)	7,700 ± 450 (16)
*Pf*KRS^N339E^	25,200 ± 2,740 (119)	26,900 ± 1,920 (55)
*Pf*KRS^F342Y^	13,600 ± 1,040 (64)	13,800 ± 1,130 (28)
*Pk*KRS^WT^	1,690 ± 134 (8)	ND^*b*^

^*a*^ Fold resistance relative to the parental WT shown in brackets.

^*b*^ ND: not determined.

In previous studies, *P. falciparum* DDD01510706-resistant clones generated through *in vitro* evolution were found to bear an S344L mutation in *Pf*KRS that conferred resistance to both DDD01510706 and cladosporin (15). *Pf*KRS^S344L^ was introduced into *P. knowlesi* parasites, also under the control of the *Pk*HSP70 promotor, and expression was confirmed through proteomics (Fig. S3). The S344L mutation led to 50- and 13-fold resistance to DDD01510706 and cladosporin, respectively (Fig. 1B and C; Table 1). The difference in resistance demonstrated by parasites expressing wild-type (WT) and mutated versions of *Pf*KRS, combined with demonstration that the S344L mutation specifically prevents inhibitor binding (15), suggests that the *P. falciparum* protein is functionally competent in *P. knowlesi*. This provides further evidence that a saturation mutagenesis library, using *P. knowlesi* as a surrogate, can provide physiologically relevant information.

### Library construction

**Fig 2:**
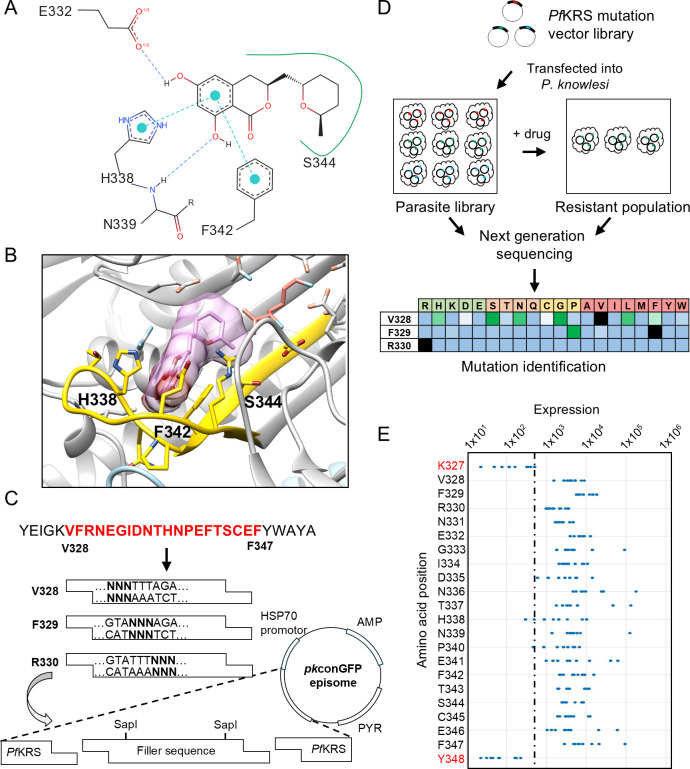
Design of vector backbone and library construction (**A**) 2D schematic and (**B**) 3D structure of the cladosporin (pink) binding site (21) highlighting residues interrogated via the mutation library (yellow; PDB ID: 4PG3). (**C**) Schematic illustrating vector construction and insert ligation to generate vector library. AMP: ampicillin resistance gene, PYR: pyrimethamine resistance gene. (**D**) Schematic outlining the final library construction, validation, and screening. (**E**) Measurement of the abundance of each sequence iteration compared with the underlying rate of sequencing error (dashed line) for the 20 amino acid positions compared with adjacent positions. Only iterations that require just a single nucleotide change from the endogenous WT sequence are included. No iterations resulting from more than one nucleotide sequencing error were identified in the two adjacent amino acids. The dashed line indicates the highest incidence of sequencing error in the two adjacent amino acid positions (327 and 348). Three nucleotide sequences were below this maximum threshold (338, CAG, Gln; 338, CGT, Arg; 340, CCG, Pro); however, these amino acids were included in the library via other synonymous nucleotide sequences.

Since *Pf*KRS is functionally competent in *P. knowlesi* and a single mutation can confer enhanced resistance above that achievable through expression of the WT enzyme, this created a window in which the impact of other resistance-conferring mutations could be profiled. Crystallographic structures of *Pf*KRS bound to DDD01510706 and cladosporin [PDB IDs: 6HCU (13) and 4PG3 (21)] were used to guide the construction of our library and led to the identification of a 20 residue stretch within the inhibitors’ binding site (V328 to F347, Fig. 2A and B) that we intended to target. This region includes residues from the class II tRNA synthetase “motif 2,” covering the adenine-binding loop, which interacts directly with the chromone and isocoumarin moieties of DDD01510706 and cladosporin, respectively, and forms part of the KRS homomeric interface (21, 22). It also includes a strand that forms part of the base of the binding pocket and leads to the lysine site, regions that have been implicated in cladosporin selectivity (22). Other potential targets included the regions around the ATP-binding residue E500, where V501, L502, and N503 show exclusive (but weak) interactions with our screening compounds, or around G534 to R559, which has similar properties. While those sites are equally valid targets to search for resistance mutations, the presence of the known resistance mutation S344L (15) between V328 and F347 provides a useful positive control, making this region the ideal choice here. Altogether, our selection represents a good basis for our library by including sites where mutations could influence binding, stability, and ultimately potency via a range of structural effects, offering a broad assessment of the assay’s ability to identify novel resistance-conferring mutations within a specific region.

The amino acid (but not nucleotide) sequence of the selected 20-amino acid span is identical between *P. falciparum* and *P. knowlesi* and incorporates the majority of the compound-binding site (within 7 Å of inhibitors; Fig. 2A and B; Fig. S1). To create the initial plasmid library, the full length *Pf*KRS gene bearing double SapI restriction sites between nucleotides encoding V328 and F347 was introduced into our non-integrating *P. knowlesi* pkconGFP expression vector (16) (Fig. 2C). We opted to introduce the desired mutations into this construct via annealed oligonucleotides, rather than employing an alternative approach such as error-prone PCR. Our reasoning was that while error-prone PCR may require less upstream work and may permit profiling of larger targets, a major drawback would be the unlikelihood of generating all conceivable mutations via this random approach. Thus, 20 sets of overlapping oligonucleotide pairs were annealed and ligated into the SapI digested vector (Fig. 2C; Table S1). Each oligonucleotide set consisted of a degenerate sequence (“NNN,” also referred as wobble bases) at one of the 20 amino acid positions allowing encoding of all possible amino acids at these sites. This vector library was transfected into blood-stage *P. knowlesi* and maintained as episomes through selection with pyrimethamine.

The workflow for the ResMAP library construction and screening is outlined in Fig. 2D. Prior to compound screening, the coverage of our transfected *Pf*KRS mutation library was established. To confirm the presence of all possible mutated amino acids within this region, genomic DNA was isolated from the library, and *Pf*KRS-specific primers were used to amplify the region of interest (Fig. S4). The resulting PCR fragments were then analyzed by next-generation sequencing (NGS) and reads aligned to the *Pf*KRS^WT^ sequence. Reads associated with each sequence iteration were counted using a customized Python script. This analysis included the amino acids flanking the region of interest (327 and 348). Reads for these adjacent amino acid positions should only contain the WT sequence, allowing the NGS sequencing error rate to be calculated. This analysis revealed that, accepting a maximum sequencing error rate of 0.2%, >99.8% of all desired sequences were represented (Fig. 2E; Table S2). Nevertheless, the amino acid substitutions that were below this error threshold were included via other synonymous nucleotide sequences. This means that 100% of all amino acid substitutions (380 amino acid substitutions) were present in our mutation library confirming complete saturation of the region when compared to adjacent amino acids.

### Drug selection of the *Pf*KRS mutation library

Having confirmed that all desired KRS mutations were represented in our library, we next selected the parasite library with the established KRS inhibitors, DDD01510706 and cladosporin. Our aim in screening was to discriminate between parasites expressing *Pf*KRS^WT^ and those expressing *Pf*KRS bearing resistance-conferring mutations. Since expression of *Pf*KRS^WT^ results in approximately fivefold resistance to specific KRS inhibitors, we selected our mutation library with inhibitor concentrations equivalent to 25× their established EC_50_ values. Libraries were selected with cladosporin and DDD01510706 for 21 days. Drug selection had minimal impact on parasite library growth (Fig. S5). Genomic (and episomal) DNA was isolated from “resistant” parasites, and the *Pf*KRS region of interest was amplified by PCR and analyzed by NGS. Reads originating from each sequence iteration were quantified to identify the enrichment of specific *Pf*KRS mutations (Fig. 2D).

**Fig 3:**
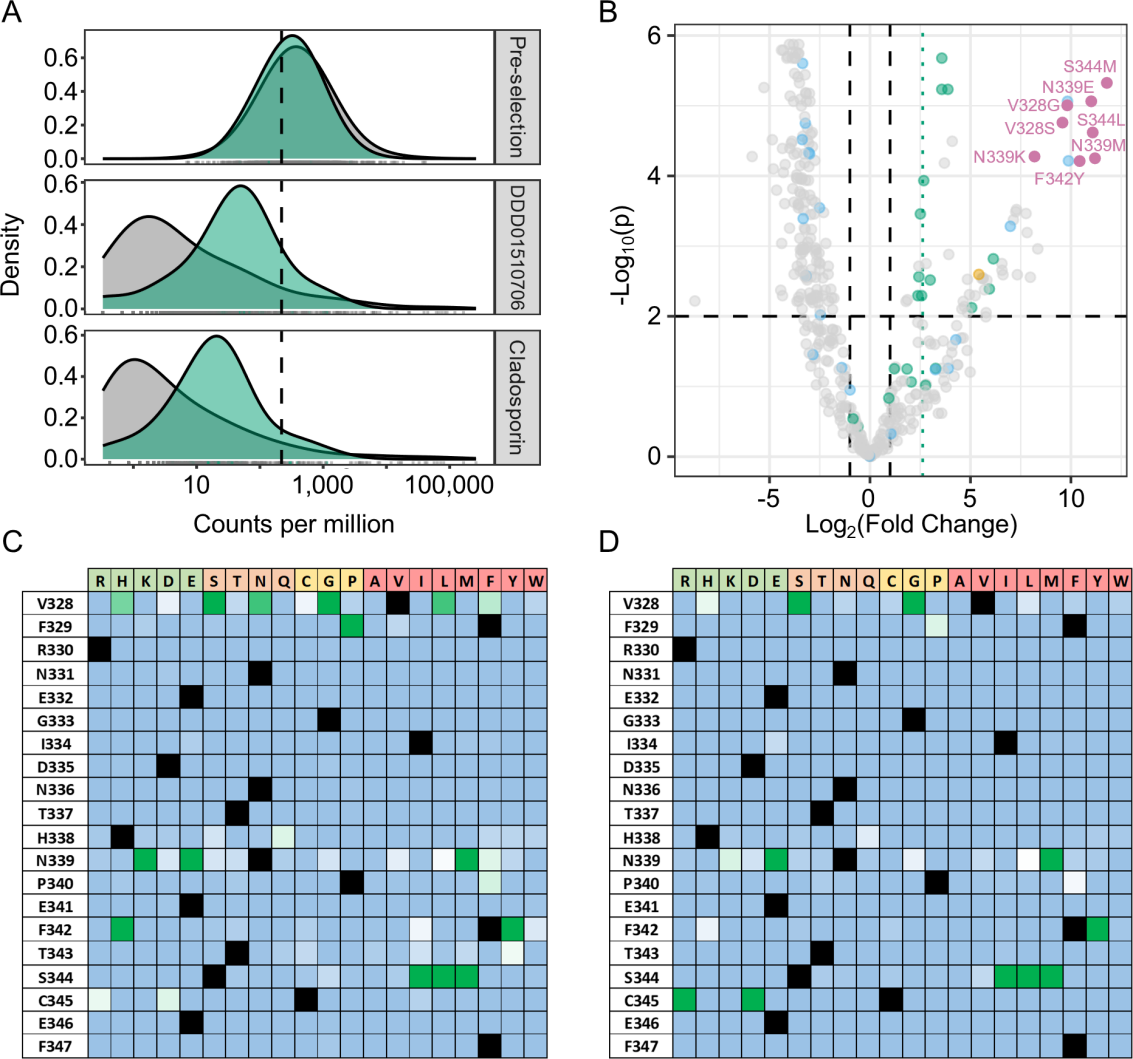
Mutation library screens with DDD01510706 and cladosporin. (**A**) Library population of synonymous (green) and non-synonymous variants (gray), pre- and post-selection with both compounds. The dashed line indicates median read counts per million for all pre-selection variants. (**B**) Volcano plot illustrating the effect of compound treatment on *Pf*KRS mutant populations. Log_2_(Fold Change): Log fold change of mutant sequence abundance post- vs pre-selection calculated. -Log_10_(p): -Log_10_ *P*-value. KRS variants are colored according to mutation type: WT (orange), synonymous (green), terminating codons (blue), missense mutations (gray), and highly significant resistance mutants (pink). Heat-maps highlighting the enrichment of resistance-conferring mutations following selection with (**C**) DDD01510706 and (**D**) cladosporin. Black squares: WT amino acid; green squares: mutation confers resistance; white squares: mutation is tolerated but provides no advantage under compound selection; light blue: mutation is not tolerated and/or provides no advantage under compound selection. Amino acids are grouped and colored in physicochemical sets: charged (green), polar (orange), hydrophobic (red), and the distinctive special cases of cysteine, glycine, and proline are highlighted together (yellow).

Following selection with DDD01510706, parasites expressing *Pf*KRS bearing 24 specific amino acid substitutions (out of a possible 380 non-terminating substitutions) were enriched ≥2 fold, while selection with cladosporin enriched 15 specific substitutions (Fig. 3; Tables S2 to S5). In line with expectations, the post-selection populations showed a stronger depletion of mutant sequences compared to synonymous sequences (Fig. 3A). Additionally, the decreased abundance of most alleles, including synonymous variants, is due to the post-selection populations being dominated by a few high-prevalence *Pf*KRS variants, resulting in a long-tailed non-synonymous distribution in these samples. Analysis with edgeR (23) confirmed the robustness of these hits, reporting a significance of *P* < 0.0001 for these resistance candidates (Fig. 3B; Table S5). Mutation of four specific amino acid positions was strongly associated with resistance, namely V328, N339, F342, and S344. The identification of S344 in library selections with cladosporin and DDD01510706 is particularly noteworthy since an S344L mutation was associated with resistance to both compounds following *in vitro* evolution of resistance (15). Earlier reports, which validated mutations derived from comparison to resistant yeast (*Saccharomyces cerevisiae*), also implicated positions S344 and V328 in inhibitor resistance (14), lending further credibility to the screen. Since the library is saturated at the codon level, many single amino acid mutations are replicated with distinguishable genotypes. For instance, the S344L point mutation is encoded via six synonymous codons with significant post-selection enrichment observed for five of the six codons (Fig. S6).

**Fig 4:**
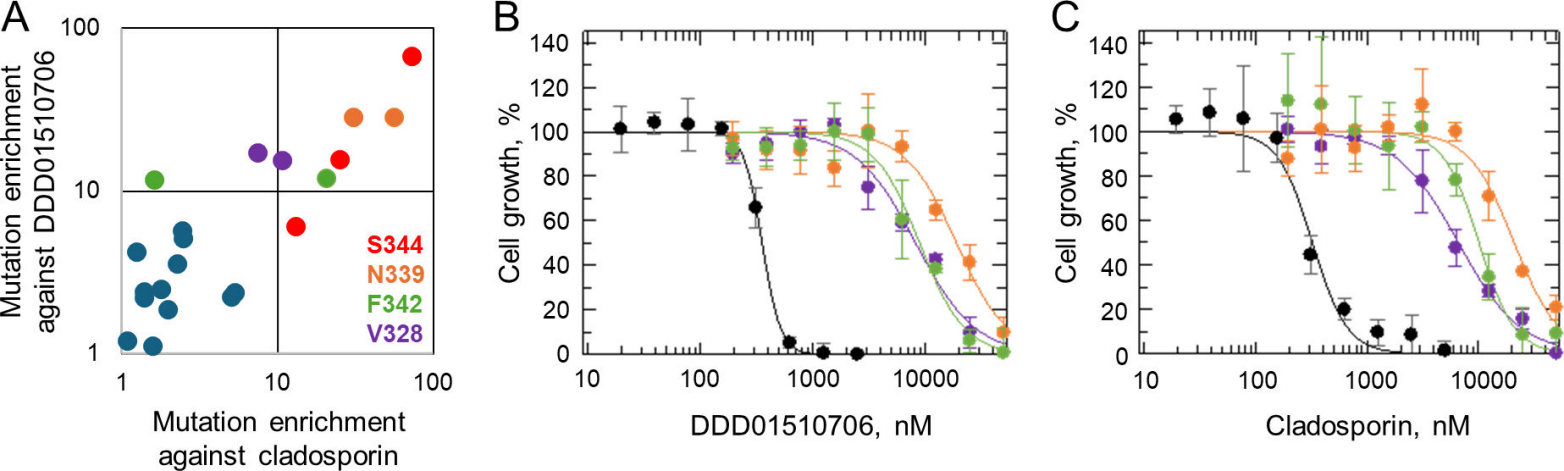
Validation of potential resistance-conferring mutations. (**A**) Comparison of resistance mutations enriched following a screening of the mutation library with cladopsorin vs DDD01510706. Nine non-terminating substitutions enriched >10-fold were identified in both compound screens. (**B**) Dose-response curves of DDD01510706 against WT (black) and *P. knowlesi* expressing *Pf*KRS^V328S^ (purple), *Pf*KRS^N339E^ (orange), and *Pf*KRS^F342Y^ (green). EC_50_ values: WT: 355 ± 9 nM, *Pf*KRS^V328S^: 8,230 ± 966 nM, *Pf*KRS^N339E^: 19,000 ± 2,150 nM, and *Pf*KRS^F342Y^: 8,910 ± 777 nM. (**C**) Dose-response curves of cladosporin against WT (black) and parasites expressing *Pf*KRS^V328S^ (purple), *Pf*KRS^N339E^ (orange), or *Pf*KRS^F342Y^ (green). EC_50_ values: WT: 324 ± 30 nM, *Pf*KRS^V328S^: 6,570 ± 382 nM, *Pf*KRS^N339E^: 20,900 ± 2,400 nM, and *Pf*KRS^F342Y^: 10,100 ± 967 nM. Representative dose-response curves consisting of three technical replicates ±SD shown.

Despite clear structural differences between cladosporin and DDD01510706 (Fig. 1A), resistance to both compounds was associated with broadly similar mutations (Fig. 3C, D and 4A; Table S5). Interestingly, several mutations led to loss of the modest resistance (approximately fivefold) conferred by expression of *Pf*KRS^WT^ in *P. knowlesi* suggesting that these mutations ablate the activity of this acyl tRNA synthetase, perhaps by interfering with ATP binding or causing the protein to misfold thus preventing inhibitor binding (Fig. 3). Reassuringly, most deleterious mutations were found to introduce premature terminating codons or missense mutations into *Pf*KRS, while synonymous mutations display a similar survival as the endogenous residue sequence. These data confirm that this screen can discriminate mutations that are detrimental to protein function from those that are tolerated but do not confer resistance, reinforcing our confidence in our screening strategy.

### Validation of resistance-conferring mutations identified through library screening

Next, we sought to validate a cohort of mutations identified through screening of our library. Three amino acids positions (V328, N339, and F342), not previously associated with KRS inhibitor resistance following *in vitro* selection, were selected for confirmatory studies (Fig. 4A). Single mutations at each position (V328S, N339E, and F342Y), identified among the top “hits” of the screen, were investigated in detail (Fig. 4A; Tables S2 to S5). All codons capable of encoding these specific amino acid changes were enriched in the course of library screening, providing additional confidence in the direct role of these mutations in compound resistance (Fig. S5). To assess the direct and specific role of these individual mutations in resistance to cladosporin and DDD01510706 individual *P. knowlesi* cell lines expressing *Pf*KRS^V328S^, *Pf*KRS^N339E^, and *Pf*KRS^F342Y^ were generated.

Label-free proteomics confirmed that our transgenic *P. knowlesi* cell lines were expressing *Pf*KRS^V328S^, *Pf*KRS^N339E^, and *Pf*KRS^F342Y^ (Fig. S3). Next, the susceptibility of these *Pf*KRS expressing cell lines to cladosporin and DDD01510706 was established (Fig. 4B and C; Table 1). Cell lines expressing *Pf*KRS with N339E, V328S, and F342Y demonstrated significant levels of resistance to both cladosporin and DDD01510706 with 15- and 119-fold shifts in susceptibility relative to WT parasites and 2- and 30-fold shifts in susceptibility compared to parasites expressing *Pf*KRS^WT^. Interestingly, the F342Y mutation results from a single nucleotide change yet has never been observed in drug-resistant lines selected through conventional *in vitro* evolution (14, 15, 24). Collectively, these data implicate positions 328, 339, 342, and 344 as pivotal in drug resistance and validate the role of the mutations selected in our mutation library in KRS inhibitor drug resistance.

### Exploring the impact of enriched mutations on compound binding

**Fig 5:**
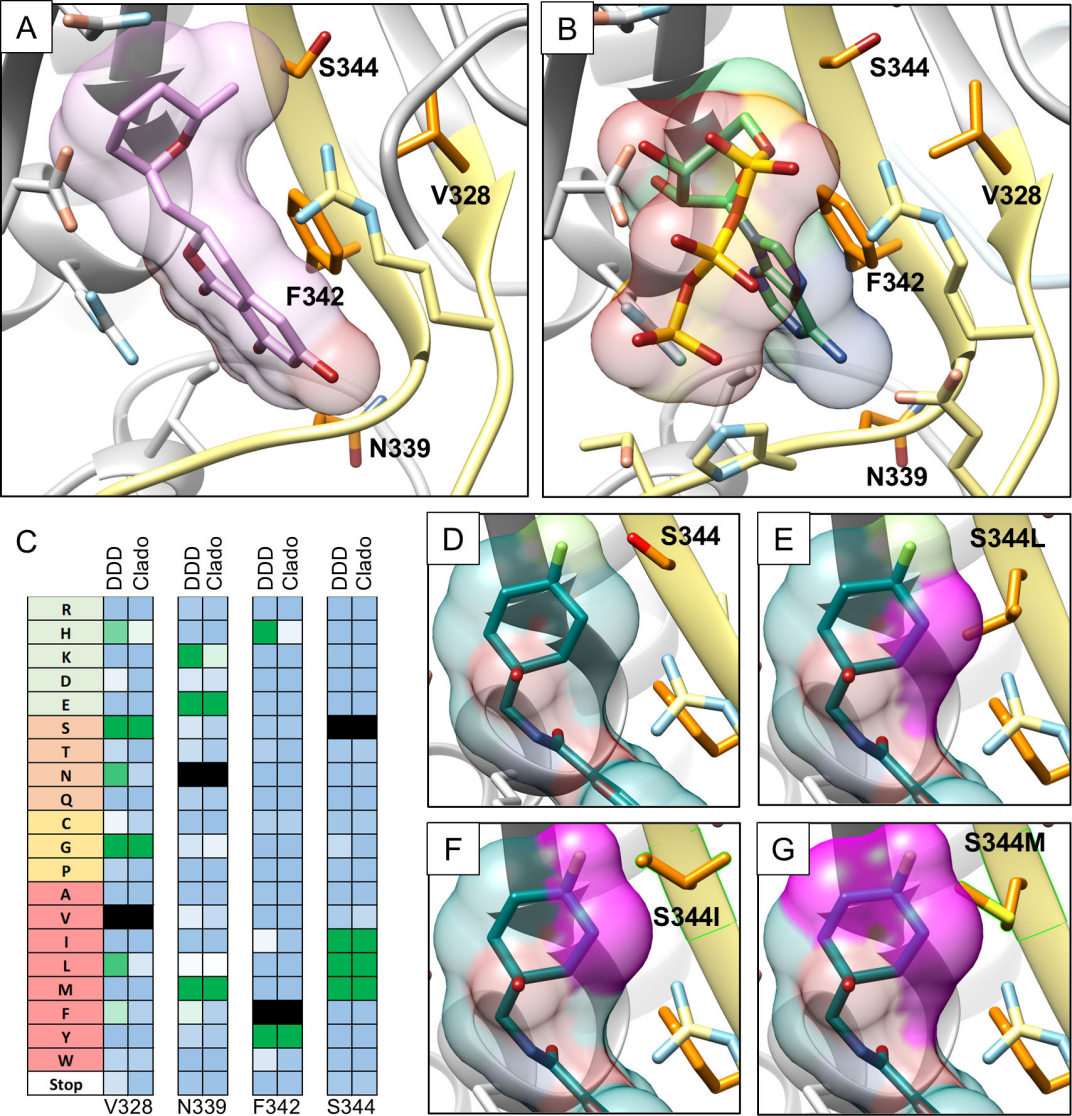
Structural rationale for resistance conferred by mutations identified through deep mutagenesis screening four sites where resistance mutations (orange) were identified among the targeted residues (yellow) shown on (**A**) the cladosporin bound structure (PDB ID: 4PG3) and (**B**) the ATP bound model. (**C**) Heat-maps of selected amino acid positions showing mutation profiles with resistance mutations against both compounds. DDD: DDD01510706; Clado: cladosporin. (**D–G**) Structural models of S344 and resistance mutants highlighting steric clashes introduced with DDD01510706 (magenta; models derived from PDB ID: 6HCU).

In the 3D structure of the inhibitor-binding site, three of the four resistance sites (V328, F342, and S344) define a clear resistance hotspot at the base of the binding site, while the fourth (N339) is located just outside of this cluster in the adenine-binding loop (Fig. 5A). Each resistance hot spot exhibited a unique mutation profile that correlated well with the known binding modes of both cladosporin and DDD01510706 and how these inhibitors orientate with respect to ATP. The most constrained site with validated resistance mutants is F342, which is π-stacked with the adenine ring of ATP (Fig. 5B). The selected F342H and F342Y mutants are among the few mutations that can possibly maintain this interaction, thereby offering the potential to preferentially disrupt binding of the inhibitors through altered interaction with the isocoumarin and chromone moieties of cladosporin and DDD01510706, respectively. In contrast to these subtle substitutions, mutations identified at position V328 were highly varied, correlating with this amino acid’s second shell position with respect to the inhibitors, where V328 interacts with the posterior face of F342. At V328, a broad range of amino acid substitutions were tolerated and enriched in our screens included the smaller (V328G and V328S), larger (V328L and V328F), as well as polar residues (V328N and V328H). These data suggest a particular plasticity at V328 that can be exploited to evade interactions with these inhibitors. S344-resistant mutants are all to hydrophobic residues that are larger than WT (S344I, S344L, and S344M). Modeling suggests that these mutations introduce steric clashes with both inhibitors and potentially disrupt ATP binding at the ribose ring, correlated with relative enrichment of each mutant (i.e., S344M >> S344L > S344I; Fig. 5C through G). Adjacent to this cluster, N339 was the fourth site with significant resistance potential identified through screening. This residue is located in the adenine binding loop between the beta-strands of the residues at the base of the pocket. In this case, both inhibitors interact with the backbone carbonyl of N339, and therefore, the resistance mutations (N339E and N339M) must act by modifying loop dynamics or quaternary interactions. N339 is involved in a few intra-molecular interactions that are potentially important for dimerization, including a hydrogen bond to T578 and multiple contacts with dimer interface residues (Fig. S7). Conversely, the main cold spot is located at the loop element away from the inhibitor binding site but near the ATP phosphate, including R330.

## DISCUSSION

Here, we describe the development of ResMAP, a deep mutagenesis screening platform capable of comprehensively profiling resistance-conferring, target-specific mutations. As proof-of-concept for this approach, we investigated resistance profiles associated with the *Pf*KRS-specific inhibitors cladosporin and DDD01510706. Screening of our mutation library revealed several new mutations not previously associated with cladosporin or DDD01510706 resistance. The relatively low transfection efficiency of *P. falciparum* precluded the development of the methodology in this parasite; however, our data indicate that *P. knowlesi* represents a highly effective surrogate. The superior transfection efficiency of *P. knowlesi* combined with its ability to express *P. falciparum* proteins enabled the generation of a complex library. Until such time that strategies to improve the transfection efficiency of *P. falciparum* emerge, *P. knowlesi* represents an effective model. Using the *P. knowlesi* model, we believe that the ResMAP approach can be readily applied to the study of other compound-target combinations. Indeed, the platform could be extended to the study of drug resistance in other *Plasmodium spp*.

Arguably, the most powerful application of ResMAP would be to support on-going target-focused drug discovery projects. Library screens carried out with diverse chemotypes have the potential to produce data capable of guiding rational drug design. The identification of amino acids within the inhibitor-binding site and with a high tolerance for mutation, particularly those associated with the highest levels of resistance, could facilitate the design and selection of inhibitors with reduced resistance susceptibility. While it may not be possible to design “irresistible” inhibitors that remain selective for a single target, previous studies have shown that it is possible to design compounds that avoid the most serious resistance-conferring mutations (6, 25). Given the paucity of robustly validated drug targets in *Plasmodium spp*., ResMAP could also be applied to the study of resistance-compromised molecular targets, such as dihydrofolate reductase (*Pf*DHFR) (26), offering the opportunity to guide the development of novel inhibitors with reduced resistance potential. The fact that experimental design and the ability to fully exploit the results of screening require access to high-quality structural models (X-ray, NMR, or cryo-EM structures) of the target of interest could be considered a limitation of ResMAP. Particularly since recombinant expression and crystallization of *Plasmodium* proteins is notoriously difficult. However, machine-learning methods such as AlphaFold 2 (AF2) have dramatically improved protein structure prediction, with accuracy approaching that of experimentally determined structures (27). These AI-generated models can now be used to accurately predict the binding modes of inhibitors (28). Once in lead optimization, ResMAP could be a useful tool for candidate selection providing an additional metric for comparing compounds. Thus, ResMAP in concert with AF2 offers an opportunity to probe the resistance landscape of previously unexplored drug targets with the realistic hope of identifying key residues involved in drug binding.

Data derived from ResMAP screens could potentially be used to monitor or indeed predict clinical resistance to specific developmental drugs. Clinical isolate sequencing data could be profiled for the specific mutations conferring high-level resistance while having a limited impact on parasite fitness. Such information could be used to predict the likelihood of naturally occurring resistance within existing parasite populations to drugs in development. Alternatively, mutations of particular concern (high resistance/high parasite fitness) identified through screening could be used as markers for the emergence of resistance to specific drugs once they are in the clinic. At present, *Pf*KRS-targeting drugs are not used to treat malaria. Nevertheless, we can use *P. falciparum* clinical isolate data to investigate the potential for mutations identified in screens with cladosporin or DDD01510706 to pre-exist in the parasite population. For example, we explored the genetic diversity of *Pf*KRS using the MalariaGEN Pf7 database (29) to provide a baseline level of genetic variance. Profiling of this database, composed of >20,000 sequenced *P. falciparum* genomes isolated from infected patients, indicated that there were at least 267 positions of variability within this gene, most encoding missense variants (Table S6). Based on the binding pocket of both KRS inhibitors, five mutations within our predicted inhibitor-binding site and profiled in our ResMAP library were identified. Fortunately, none of these mutations correlated with the mutations associated with high-level resistance that were identified in our screens. Early notification of the emergence of mutations providing high-level resistance could offer opportunities for interventions to minimize the spread of resistant infections.

While the identification of resistance-conferring mutations is important, the ability to assess the fitness cost associated with specific mutations is vital to understand their potential clinical impact. Adapting our current screening strategy by increasing the duration of drug selection should allow mutations conferring the best combination of resistance and fitness to be readily identified. Indeed, the selection of the library over a range of drug concentrations may also allow the level of resistance provided by specific mutations to be determined without the need for time-consuming validation studies. In the majority of cases, selection with target-specific inhibitors will select parasites expressing the target-bearing mutations that directly or indirectly impact inhibitor binding. However, not all enriched mutations identified through ResMAP will be related to target binding. For instance, mutations that enhance the activity of a particular target, as observed in *in vitro* selections with *Pf*CLK3 inhibitors (30), will also be identified. In contrast, mutations that require the presence of a second, compensatory mutation to achieve the highest levels of resistance (26) will not be identified via this methodology.

Here, we focused on a linear region of the established binding site of our KRS inhibitor to provide proof-of-principle for this approach. However, in future iterations of the library, we will aim to expand upon this region to cover every residue within the binding region, encompassing genetically distinct regions of the target protein. This will be achieved through the synthesis of multiple constructs, each focused on different regions of *Pf*KRS. The number of positions that can be interrogated by a single vector is limited by the length of the synthesized oligonucleotides (≤100 bp) since increased oligonucleotide size correlates to reduced production efficiency. Nevertheless, the high-ligation efficiency of these oligonucleotides facilitates the generation of multiple smaller vector libraries that, when combined, have the potential to cover an almost unlimited region of the protein. The primary challenge will be to design suitable PCR amplification reactions and analysis of the resulting next-generation sequencing. In the present study, we leveraged 150 bp paired-end sequencing reads that covered the entirety of our 20 aa library (the exact alignment of ≥90 bp sequences was required for each iteration in this study). Increasing the library size would require an alternative approach for the final data analysis; however, established tools, such as DiMSum, should be suited for this (31). One solution would be to add unique barcodes to each oligonucleotide insert of the vector library so that these sequences can be discriminated from the endogenous (WT) sequence in the sequencing data. Alternatively, sequence analysis could focus sequentially on each 3 bp amino acid position. This would enable analysis of libraries of unlimited size, and while the background sequencing error would increase the number of false positives, this could be mitigated with more stringent sequence read alignment prior to counting iterations. Finally, although alternative techniques, such as error-prone PCR, would allow faster generation of mutation libraries, the design of ResMAP results in the comprehensive inclusion of all mutations, with a relatively even distribution of non-synonymous changes across all positions.

Interestingly, neither of the single mutations in the V328Q/S344T double mutant, previously shown to confer cladosporin resistance, were enriched in our screens (14). Cladosporin, a fungal secondary metabolite, was originally found to have potent activity against blood-stage *P. falciparum* following a screen of natural products by the Winzeler group (32). In a subsequent screen of positive hits, they then demonstrated activity against *Plasmodium yoelii* liver stages (33). Target deconvolution studies using yeast and *P. falciparum* demonstrated specific inhibition of protein synthesis through direct targeting of KRS (14). Based on alignments of the cladosporin-binding sites of *P. falciparum* and the naturally resistant *S. cerevisiae*, only two amino acids (V328 and S344) were divergent, and thus, mutations in these positions were predicted to also provide resistance in *Plasmodium*. Indeed, inclusion led to ~33-fold resistance in a recombinant *Pf*KRS protein assay following “yeastification” of these amino acids; however, the mutations have not been validated independently of each other or identified directly in *Plasmodium* via *in vitro* resistance selection experiments (14).

Another unexpected finding was the enrichment of specific stop codons at positions F329, H338, and T343. These stop codons would likely yield non-functional protein and would be unlikely to confer resistance outside our episomal expression design, so they were excluded from validation. Reassuringly, only 3 out of 60 stop mutants in the library were enriched post-selection, suggesting a false discovery rate of about 5%. However, the significant and consistent enrichments in both treatments indicate a genuine biological effect. One possibility is that truncated KRS might sequester inhibitors when highly expressed, a condition that could be met in random individuals within pre-selection mutant sub-populations. Alternatively, the selection of these stop codons could be due to a relatively low signal-to-noise ratio leading to the selection of false positives, although no other codons appear to have been erroneously selected. Another possibility is that these stop codons were read-through, substituting an alternative amino acid, such as selenocysteine, explaining the absence of other enriched mutations at these sites. Although rare, this process is known to occur in *Plasmodium* (34, 35). Regardless of the precise mechanism, since this was observed in both inhibitor screens, it is likely a random effect related to stop codons in the pre-selection population. While these findings highlight a potential for relatively rare false positives, our detection of S344L and validation of three out of three additional novel positions suggests this is not a widespread issue, and the screen is fit for purpose. Nevertheless, understanding this further would be valuable, and future work could involve additional replicates to confirm if this behavior applies to random stop mutants, discounting a deterministic phenomenon. Finally, we constructed our library using episomal expression of *Pf*KRS to maximize library complexity, ensuring all conceivable mutations are included. However, our data cannot discount the possibility that variable expression levels of individually mutated enzymes may also impact selective enrichment (Fig. S3). Given the complexity of the library, it is entirely possible that multiple parasite subpopulations could be transfected with the same episome thus normalizing the level of enrichment assigned to a specific mutation. Since such a high transfection efficiency is achievable in *P. knowlesi*, future work will investigate the use of integrating plasmids to stabilize expression levels throughout the library. This will provide greater clarity on the impact of the putative resistance mutation vs just target overexpression on drug resistance.

In conclusion, the ability to identify and profile all conceivable resistance-conferring mutations as part of a drug discovery program provides an opportunity to design compounds that can confound the emergence of drug resistance. Here, we have provided proof-of-concept for a method to rapidly and comprehensively profile all target-associated, resistance-conferring mutations. Profiling of all possible resistance-conferring mutations can inform the development of resistance-resilient inhibitors as well as providing tools to survey resistance in the future.

## MATERIALS AND METHODS

### Parasite culture

*P. knowlesi* A1–H1 strain was kindly provided by Dr Robert Moon (LSHTM). Blood-stage parasites were cultured in RPMI 1640 (HEPES Modification with 25 mM HEPES, without L-glutamine) with the following additions: 2.3 g L^−1^ sodium bicarbonate, 2 g L^−1^ dextrose, 0.05 g L^−1^ hypoxanthine, 5 g L^−1^ Albumax II, 0.3 g L^−1^ L-glutamine, 10% (vol/vol) horse serum, sterile filtered, and stored at 4°C. Parasites were grown in fresh blood (<2 weeks old) at 37°C in a gas mixture of 96% N_2_, 3% CO_2_, and 1% O_2_.

### Blood collection

Blood was collected by venous puncture from anonymous volunteers at the University of Dundee. Venous blood (8 mL) was collected in K_3_EDTA vacuette tubes (Greiner bio-one) with red blood cells (RBCs) washed with RPMI 1640, stored at 4°C and used within 2 weeks. Ethical approval for blood collection protocols and donor consent frameworks were approved by the University of Dundee Research Ethics Committee (Reference 19/88).

### Compound 2 synthesis

Compound 2 is a PKG (cGMP-Dependent Protein Kinase) inhibitor used to synchronize *Plasmodium* parasites for transfection (36). Synthesis of 15 mg of 4-{7-[(dimethylamino)methyl]−2-(4-fluorophenyl)imidazo (1,2-α)pyridine-3-yl}pyrimidin-2-amine [compound 2; CAS 480453–85-8 (37)] was prepared in five steps from 1-(4-fluorophenyl)−2-(2-(methylthio)pyrimidin-4-yl)ethan-1-one (217661–99-9, Apollo Scientific Ltd) following a reported synthetic route (38). Analytical data for compound 2 were in good agreement with that reported previously (38). High-performance liquid chromatography (HPLC) analysis demonstrated that the compound was >95% pure.

### *P. knowlesi* drug susceptibility assays

Dose-response assays were performed as previously described with minor changes (39). Non-synchronous *P. knowlesi* (1% parasitemia and 1% hematocrit) was exposed to test compound in 96-well plates. Untreated parasites and parasites treated with pyrimethamine (100 nM) were used to establish maximum and minimum growth parameters, respectively. Parasites were incubated for 54 h; plates were frozen overnight at −20°C. Following plate thawing, SYBR green I (ThermoFisher) diluted 1:5,000 in Lysis Buffer A [20  mM Tris, 5  mM EDTA, 0.008% (wt/vol) saponin, 0.08% (vol/vol) Triton X-100, and pH 7.5] was added to each well (100 µL) and then incubated for 1 h in the dark at room temperature (RT). Plates were read using a microplate reader (Tecan Infinite Pro 200) at 490 nm excitation and 520 nm emission. Data were analyzed using the two-parameter equation in GraFit (Erithacus Software).

### Generation of the *P. knowlesi* KRS overexpression vector

We adapted the pkconGFPp230p vector by removing the integrating p230p locus (located between EcoRI and XhoI) and replacing it with a filler sequence (16). The full coding sequence of *Pk*KRS (PKNH_1250800) was amplified from *P. knowlesi* A1–H1 genomic DNA using primers PkKRSOE_f and PkKRSOE_r (Table S1). The PCR amplified product was ligated into pkconGFP via XmaI and SacII restriction sites (replacing eGFP). The resulting plasmid was Sanger sequenced in-house prior to transfection (MRC PPU DNA Sequencing Facility, University of Dundee).

### Mutation library construction

The full *Pf*KRS coding sequence (PF3D7_135010) with introns removed was synthesized (GeneArt) and ligated into our adapted pkconGFP vector via XmaI and SacII restriction sites. The synthesized gene maintained a stuffer region that was removable by double SapI digestion allowing subsequent ligation of annealed oligonucleotide pairs (ThermoFisher Scientific) encoding specific mutations. Each oligonucleotide pair was focused on a single amino acid position and encompassed “*NNN*” sites allowing all conceivable codons to be represented. Oligonucleotide pairs were annealed by heating at 95°C for 2 min followed by cooling at RT. Annealing oligonucleotides generated overhangs that were compatible with the SapI-digested pkconGFP-*Pf*KRS vector enabling ligation. Twenty annealed oligonucleotide pairs were ligated into SapI-digested pkconGFP-*Pf*KRS to encode mutations at the 20 amino acid positions selected for interrogation via library screening. Analysis of the nucleotide ratios revealed a slight bias, introduced at the oligo synthesis stage, for T and A nucleotides (Fig. S8), indicating that the library moderately overrepresents amino acids encoded with these nucleotides.

### Transfection of *P. knowlesi*

Transfection of the vector library into *P. knowlesi* parasites was achieved as previously described (36). Briefly, schizonts were enriched from 25 mL cultures (~8% parasitemia; 2% hematocrit) following elution from a density gradient interface (55% Nycodenz column), as previously described (36). Enriched schizonts were incubated with 1 µM compound 2 for 2 h. Compound 2-treated schizonts were pelleted by centrifugation (900 g, RT, 5 min) and resuspended in 1 mL media. Schizonts (1–2 × 10^8^ cells) were transfected with 10 µg purified vector library using a 4D Amaxa nucleofector (Pulse code: FP 158; Solution: Primary Cell P3, Volume: 100 µL), then allowed to reinvade fresh RBCs (<3 days old) and transferred to 10 mL flasks. Twenty-four hours following transfection, parasites were selected with 100 nM PYR for 4 days. Fresh media and drug were applied daily. All parasites surviving the 6-day treatment with PYR were pooled to form the mutation library. Ten days post transfection, an aliquot of the parasite population was harvested for genomic DNA extraction. Since the library is maintained as episomes, PYR selection was maintained prior to test-drug selection.

### Library selection

Compound screening was performed with 50 mL culture of the mutation library. Each library was screened with 10 µM compound (equivalent to ~25× the established EC_50_). The library was passaged every ~2 days with fresh media and selection compound; however, it was not maintained in pyrimethamine following the commencement of the screening. After 21 days of selection, genomic DNA was isolated from resistant parasites ready for downstream analysis.

### Harvesting genomic DNA

Blood stage parasites (parasitemia ~5%, 2% hematocrit, 50 mL culture, and ~10^8^ parasites) were washed with RPMI then incubated in 0.015% (wt/vol) saponin for 10 min. Samples were centrifuged at 1,800 *g* for 5 min (full acceleration and medium deceleration). The purified parasite pellet was washed thrice with PBS then resuspended in 500 µL DNA lysis buffer [10 mM Tris-HCl pH 8, 100 mM NaCl, 25 mM EDTA, 0.1 mg mL^−1^ proteinase K, and 0.5% (wt/vol) SDS]. Samples were incubated overnight at 56°C, then DNA was isolated via one wash with 1 vol of phenol-chloroform and one wash with 1 vol of chloroform. Two volumes of 100% ethanol were added, and samples were centrifuged for 15 min at 4°C. Supernatant was removed, and DNA pellet was resuspended in 10 µL Tris buffer.

### Next-generation sequencing of selected library populations

The region of the episomal *Pf*KRS containing the amino acid changes was amplified with *Pf*KRS specific primers, PfKRSmutlibPCR_f, and PfKRSmutlibPCR_r, using a high-fidelity PCR polymerase (Fig. S4; Table S1). The primers (ThermoFisher Scientific) were designed so that the mutation library region would be completely encompassed within one of the 150 bp paired end sequencing reads (Fig. S9). Purified PCR products were then sequenced using a DNB-seq machine at Beijing Genomics Institute (BGI, Hong Kong). Paired-end reads (PCR-free library construction; PE150) were aligned to a reference sequence using Bowtie2 (with the settings: --very-sensitive-local) of the same region of *Pf*KRS but with “N” in the 60 bases consisting of the library so that there was no alignment bias. The aligned files were then manipulated using SAMtools (40). A customized script was used to count each iteration of the *Pf*KRS sequence and is available on GitHub from https://github.com/stuartmac/ResMAP. Excel was then used to (i) to confirm that all substitutions were present in the library and (ii) to compare the unselected library with selected libraries. For each data set, the number of a particular sequence was normalized against the total number of sequences for that screen to give a percentage of each subpopulation. To calculate the baseline error rate for the unselected library, these normalized values were compared against the equivalent values for the amino acids immediately adjacent to the 20 amino acids of interest (positions 327 and 348). There were no detected PCR and/or sequencing errors consisting of more than one substitution in these adjacent positions. A similar analysis was performed for the selected library screens; however, the normalized values were then compared against the unselected library to measure the enrichment of each population.


$$Enrichment=\frac{\left(\frac{R^{s}}{TR^{s}}\right)}{\left(\frac{R^{us}}{TR^{us}}\right)}$$

Where *R* = read count for a given amino acid sequence, TR = total read count for all sequences, *s* = selected library, and us = unselected library.

### Analysis of enrichment using edgeR

A preliminary analysis indicated that the selection of the library with cladosporin and DDD01510706 elicited similar mutation selections (Pearson correlation = 0.90; Fig. S10). This suggested the feasibility of treating the two treatments as replicates for a robust assessment of population changes using edgeR (23). Accordingly, normalization was performed with “normLibSizes” followed by dispersion estimation with “estimateDisp” under a design that treated both compounds as replicates. A generalized linear model framework (“glmLRT”) was applied to the normalized data. We used a likelihood ratio test for determining differential expression. We ran the analysis at both the codon and residue levels. For the residue-level analysis, we pre-processed the data to aggregate all counts of synonymous nucleotide sequences.

### Recovering overexpression vectors from compound-selected libraries

Genomic DNA was harvested from compound-selected libraries, as described above. This harvested DNA containing episomal vector DNA (2 µL; ~300 ng) was transfected into ElectroMAX DH10B *E. coli* via electroporation, as per the manufacturers’ instructions. Electroporated cells were plated onto LB-amp plates and incubated at 37°C overnight. Individual colonies were selected and grown overnight in LB-amp media, then DNA was isolated under standard conditions. The purified vector was Sanger sequenced to identify the mutation responsible for resistance. From this, vectors containing the V328S, N339E, F342Y, and S344L mutations were selected for further analysis.

### Protein quantification

Protein samples were prepared from *P. knowlesi* blood-stage parasites (10 mL, WT and transgenic). Cultures (~8% parasitemia; 2% hematocrit) were harvested by centrifugation (5 min, RT, 1,800 *g*), washed with 10 mL RPMI, prior to incubation in RPMI plus 0.015% (wt/vol) saponin for 10 min at RT. Following incubation, cell pellets were harvested by centrifugation (5 min, RT, 1,800 *g*) and washed 3× with 1× PBS. Cell pellets were lysed in Lysis Buffer [30 µL; 1× PBS with 2× Roche protease inhibitor and 1% (vol/vol) NP40], then harvested by centrifugation (15,000 *g*, 10 min at 4°C). The supernatant was transferred to a new tube with 10 µL of 4× Laemmli buffer and 2 µL of dithiothreitol (DTT, 50 mM final) then boiled for 5 min at 95°C. Samples were run for 8 min (1.5 cm) into a NuPAGE Bis–Tris 10% (wt/vol) acrylamide gel and stained with Instant Blue Coomassie stain (Abcam) for 30 min. The entire gel bands were removed and subjected to in-gel reduction with 10 mM DTT, alkylation with 50 mM iodoacetamide, and digestion with 12.5 µg mL^–1^ trypsin (Pierce) for >16 h at 37°C. Recovered tryptic peptides were then vacuum-dried prior to analysis. Liquid Chromatography with tandem mass spectrometry (LC–MS/MS) analysis using a Q Exactive Plus mass spectrometer coupled with a Dionex Ultimate 3000 RS was performed, as previously described (41).

### Molecular modeling

Crystal structures of *Pf*KRS bound to cladosporin (PDB ID: 4PG3) and DDD01510706 (PDB ID: 6HCU) were retrieved from the Protein Data Bank. Inhibitor-binding sites were visualized with UCSF Chimera, and mutant models were generated with the “swapaa” command after deleting ligands and waters. The ATP-docked model was retrieved from the Alphafill database (model: Q8IDJ8, ATP transplanted from PDB ID: 3BJU with transplant clash score = 0.2). Overall Root mean square deviation (RMSDs) between the structures were 0.704 Å (6HCU vs Q8IDJ8), suggesting that the AlphaFold/Alphafill model is reliable. Sequence alignments were generated using clustalW 2.1.

## Data Availability

The proteomic data sets were deposited to ProteomeXchange (PXD049335), and the raw counts from the next-generation sequencing is included in the supplementary information. The customized script used to count each iteration is available on GitHub from https://github.com/stuartmac/ResMAP.

## SUPPLEMENTAL MATERIAL

Supplemental Information mbio.01708-24-s0001.docx Supplemental figures and Tables S1 and S5.

Supplemental Tables mbio.01708-24-s0002.xlsx Tables S2–S4 and S6.

ASM does not own the copyrights to Supplemental Material that may be linked to, or accessed through, an article. The authors have granted ASM a non-exclusive, world-wide license to publish the Supplemental Material files. Please contact the corresponding author directly for reuse.
