# Supplementary material for: ResMAP—a saturation mutagenesis platform enabling parallel profiling of target-specific resistance-conferring mutations in Plasmodium
Source: mBio. 2024 Aug 27;15(10):e01708-24. doi: 10.1128/mbio.01708-24 (PMC11481570; doi:10.1128/mbio.01708-24)
Supplement: Supplemental Information — Supplemental figures and Tables S1 and S5. [file mbio.01708-24-s0001.docx]

**SUPPLEMENTARY INFORMATION**

**ResMAP – a saturation mutagenesis platform enabling parallel profiling of target-specific resistance conferring mutations in *Plasmodium***

Richard J. Wall^1,6,*^, Stuart A. MacGowan^2^, Irene Hallyburton^3^, Aisha J. Syed^1^, Sowmya Ajay Castro^4^, Gourav Dey^1^, Rachel Milne^1^, Stephen Patterson^1^, Jody Phelan^5^, Natalie Wiedemar^1,7^ and Susan Wyllie^1,*^

^1^Wellcome Centre for Anti-infectives Research, Division of Biological Chemistry and Drug Discovery, School of Life Sciences, University of Dundee, Dow Street, Dundee, DD1 5EH, UK

^2^Division of Computational Biology, School of Life Sciences, University of Dundee, Dow Street, Dundee, DD1 5EH, UK

^3^Drug Discovery Unit, Wellcome Centre for Anti-infectives Research, Division of Biological Chemistry and Drug Discovery, University of Dundee, Dow Street, Dundee, DD1 5EH, UK

^4^Division of Molecular Microbiology, School of Life Sciences, University of Dundee, Dundee, DD1 5EH, UK

^5^Department of Infection Biology, Faculty of Infectious and Tropical Diseases, London School of Hygiene and Tropical Medicine, London WC1E 7HT, UK

^6^Current address: Department of Infection Biology, Faculty of Infectious and Tropical Diseases, London School of Hygiene and Tropical Medicine, London WC1E 7HT, UK

^7^Current address: Institute of Parasitology, Department of Infectious Diseases and Pathobiology, Vetsuisse Faculty, University of Bern, Länggassstrasse 122, 3012, Bern, Switzerland

**Table of contents**

[Supplementary Figures 4](#_Toc168407437)

[Supplementary Figure S1: Amino acid alignment of *P. knowlesi* and *P. falciparum* KRS 4](#_Toc168407439)

[Supplementary Figure S2: Overexpression of *Pk*KRS in *P. knowlesi* 5](#_Toc168407440)

[Supplementary Figure S3: Overexpression of *Pf*KRS in *P. knowlesi* 6](#_Toc168407442)

[Supplementary Figure S4: Alignment of *P. knowlesi* and *P. falciparum* KRS genomic sequences 8](#_Toc168407443)

[Supplementary Figure S5: Growth curve of mutation library during drug selection 9](#_Toc168407444)

[Supplementary Figure S6: Specific nucleotide sequence enrichment at positions V328, N339, F342 and S344 following selection with KRS inhibitors 11](#_Toc168407445)

[Supplementary Figure S7: Structural context of N339 in KRS-DDD01510706 complex (PDB ID: 6HCU) 12](#_Toc168407446)

[Supplementary Figure S8: Nucleotide ratio content of synthesised degenerate oligonucleotides 13](#_Toc168407447)

[Supplementary Figure S9: Alignment of sequencing reads showing the mutation library region. 14](#_Toc168407448)

[Supplementary Figure S10: Correlation between post-selection read counts following treatment with both compounds 15](#_Toc168407449)

[Supplementary Tables 16](#_Toc168407450)

[Supplementary Table S1: Oligonucleotides used in this study. 16](#_Toc168407451)

[Supplementary Table S2: Read counts and library ratio for the pre-selected mutation library 18](#_Toc168407452)

[Supplementary Table S3: Read counts and library ratio following treatment with DDD01510706 normalised against pre-selected library 18](#_Toc168407453)

[Supplementary Table S4: Read counts and library ratio following treatment with cladosporin normalised against pre-selected library 18](#_Toc168407454)

[Supplementary Table S5: Selection of mutations enriched by ≥2-fold over unselected lines following selection with cladosporin or DDD01510706 18](#_Toc168407455)

[Supplementary Table S6: Genetic diversity of *Pf*KRS from clinical isolates 19](#_Toc168407456)

# **Supplementary Figures**

# *P. knowlesi* MFRYLLPILRYHKNFGSHFYKQHSFPLTILNKNKNIICPVNCKQTFTKMSEKKEHVMEGE

*P. falciparum* --MTSKSFLLSFLKYKHVNTYIFEKSFSKILKNTKKHIDCHLKSCFVTMNEKKEHVLEGE

.:* . :: .. .:: : **.: : *. *..*.******:***

*P. knowlesi* KKVPSKQQVKDKKKEEEAEIDPRLYYENRSKFVQEQKAKGINPYPHKFERTITVPEFVEK

*P. falciparum* KNKRVVNASKDKKKEEEGEVDPRLYFENRSKFIQDQKDKGINPYPHKFERTISIPEFIEK

*: : ********.*:*****:******:*:** **************::***:**

*P. knowlesi* YQNLASGEHLENTVLNVTGRIMRVSASGQKLRFFDLVGDGAKIQVLANFAFHDHTKSNFA

*P. falciparum* YKDLGNGEHLEDTILNITGRIMRVSASGQKLRFFDLVGDGEKIQVLANYSFHNHEKGNFA

*::*..*****:*:**:*********************** *******::**:* *.***

*P. knowlesi* EAYDKIRRGDIVGIVGFPGKSKKGELSIFPKETIILSPCLHMLPMKYGLKDTEIRSRQRY

*P. falciparum* ECYDKIRRGDIVGIVGFPGKSKKGELSIFPKETILLSACLHMLPMKYGLKDTEIRYRQRY

*.********************************:**.***************** ****

*P. knowlesi* LDLMINESTRSTFITRTKIINYLRNFLNDRGFIEVETPTMNLVAGGANAKPFITHHNDLD

*P. falciparum* LDLLINESSRHTFVTRTKIINFLRNFLNERGFFEVETPMMNLIAGGANARPFITHHNDLD

***:****:* **:*******:******:***:***** ***:******:**********

*P. knowlesi* LDLYLRIATELPLKMLIVGGIDKVYEIGK**VFRNEGIDNTHNPEFTSCEF**YWAYADFYDLI

*P. falciparum* LDLYLRIATELPLKMLIVGGIDKVYEIGK**VFRNEGIDNTHNPEFTSCEF**YWAYADYNDLI

*******************************************************: ***

*P. knowlesi* KWSEDFFSTLVMHLFGTYKILYNKDGPDKDPIEIDFTPPYPKVSIVEELEKLTNTKLEQP

*P. falciparum* KWSEDFFSQLVYHLFGTYKISYNKDGPENQPIEIDFTPPYPKVSIVEEIEKVTNTILEQP

******** ** ******** ******:::******************:**:*** ****

*P. knowlesi* FDSPETINKMINLIKENKIEMPNPPTAAKLLDQLASHFIENQYPNKPFFIIEHPQIMSPL

*P. falciparum* FDSNETIEKMINIIKEHKIELPNPPTAAKLLDQLASHFIENKYNDKPFFIVEHPQIMSPL

*** ***:****:***:***:********************:* :*****:*********

*P. knowlesi* AKYHRSKPGLTERLEMFICGKEVLNAYTELNDPFKQKECFSAQQKDREKGDAEAFQFDAP

*P. falciparum* AKYHRTKPGLTERLEMFICGKEVLNAYTELNDPFKQKECFKLQQKDREKGDTEAAQLDSA

*****:**********************************. *********:** *:*:.

*P. knowlesi* YCTSLEYGLPPTGGLGLGIDRITMFLTNKNCIKDVILFPTMRPAN

*P. falciparum* FCTSLEYGLPPTGGLGLGIDRITMFLTNKNSIKDVILFPTMRPAN

:*****************************.**************

Supplementary Figure S1: Amino acid alignment of *P. knowlesi* and *P. falciparum* KRS **-** Residues within a 7 angstrom distance of compound binding are highlighted green, previously identified resistance mutation is highlighted yellow (S344L) and positions included within the mutation library are in bold.


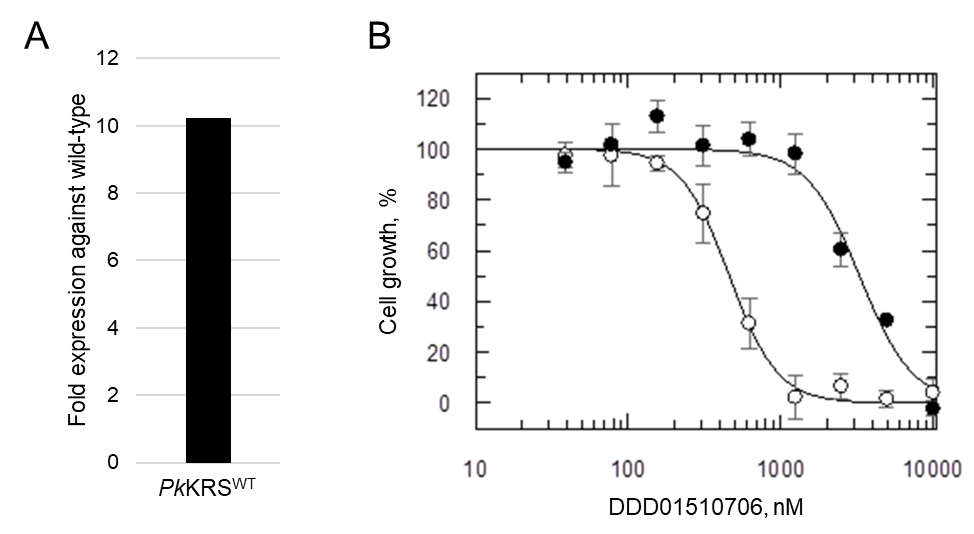


Supplementary Figure S2: Overexpression of *Pk*KRS in *P. knowlesi* – (A) Label-free quantitative proteomics confirming in 10-fold overexpression of *Pk*KRS^WT^ compared to wild-type. Protein expression was normalised against beta-actin and to WT *Pk*KRS protein levels (no episome). (B) Dose-response curves of DDD01510706 against overexpression of *Pk*KRS^WT^ (closed circles) compared to wild-type (open circles) in *P. knowlesi*. EC_50_ values were WT: 461 ± 17 nM and *Pk*KRS^WT^ overexpression: 3290 ± 297 nM. Representative experiment consisting of 3 technical replicates is shown.


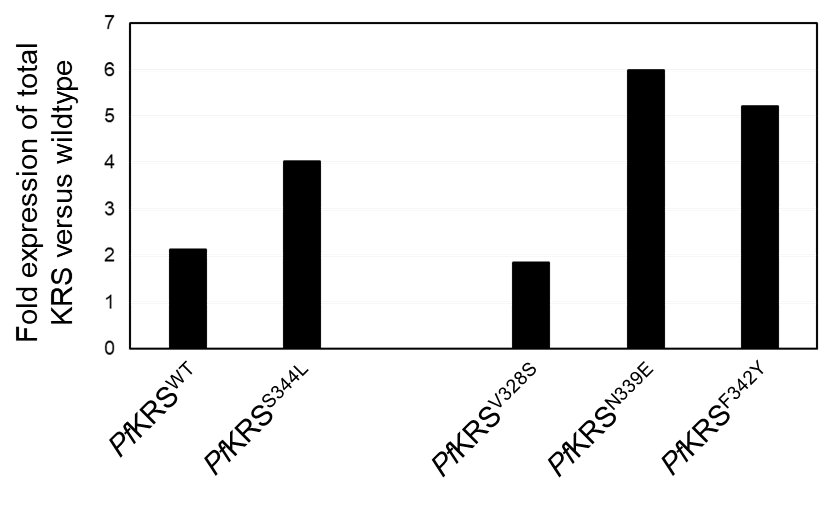


Supplementary Figure S3: Overexpression of *Pf*KRS in *P. knowlesi* – Label-free quantitative proteomics of total KRS (*Pk* and *Pf)* levels. Experiments performed in the presence of 100 nM pyrimethamine but, crucially, in the absence of KRS inhibitor thus providing only a snapshot of expression levels. Includes multiple unique *Pf*KRS peptides confirming expression. Protein expression was normalised against beta-actin and to WT *Pk*KRS protein levels (no episome).

*P. knowlesi*  ATGTTCAGATATTTACTCCCCATCCTGAGGTACCATAAAAATTTCGGAAGCCACTTTTAC

*P. falciparum*  ATGACAAGTAAGTCATTTTTATTATCCTTTTTAAAATATAAACACGTGAATACATATATT

***: .**::* * * * .:*. : *:..*::*:**: :** .*. ...*:*::

*P. knowlesi*  AAACAACATTCCTTCCCATTAACAATTCTGAACAAGAATAAAAATATCATCTGCCCGGTT

*P. falciparum*  TTTGAAAAATCATTCTCCAAAATTTTAAAAAACACAAAAAAGCACATAGATTGTCATCTA

::: **.*:**.*** *.::** ::*:.:.****..**:**..* **..: ** *. *:

*P. knowlesi*  AACTGCAAACAGACCTTCACCAAAATGAGTGAGAAAAAGGAGCATGTGATGGAGGGCGAG

*P. falciparum*  AAAAGTTG------TTTTGTCACAATGAATGAGAAAAAGGAGCACGTTCTTGAAGGCGAA

**.:* :. ** . **.*****.*************** ** .* **.*****.

*P. knowlesi*  AAGAAAGTCCCCAGCAAGCAGCAAGTCAAGGACAAGAAAAAGGAGGAAGAAGCCGAGATC

*P. falciparum*  AAGAATAAGCGAGTCGTGAATGCAAGCAAAGATAAGAAAAAAGAGGAGGAAGGTGAAGTG

*****:.: * .. *.:*.* .*. ***.** ********.*****.**** **..*

*P. knowlesi*  GACCCACGTCTATACTACGAGAACAGATCCAAGTTCGTTCAGGAACAGAAGGCCAAGGGC

*P. falciparum*  GATCCAAGATTATATTTTGAAAATCGATCCAAATTTATACAAGACCAAAAAGATAAAGGA

** ***.*: **** *: **.** .*******.** .*:**.**.**.**.*. **.**.

*P. knowlesi*  ATCAACCCTTACCCACACAAATTTGAGAGGACGATAACGGTGCCAGAATTTGTGGAAAAG

*P. falciparum*  ATCAACCCTTATCCACACAAATTTGAGAGGACAATAAGTATTCCTGAGTTTATTGAGAAA

*********** ********************.**** .* **:**.***.* **.**.

*P. knowlesi*  TACCAAAACCTAGCCAGCGGAGAGCACCTCGAAAATACCGTTCTAAATGTGACTGGAAGA

*P. falciparum*  TATAAAGATTTAGGTAATGGGGAACATTTAGAAGATACCATATTAAATATTACCGGTCGT

** .**.* *** *. **.**.** *.***.*****.*: *****.* ** **:.*:

*P. knowlesi*  ATAATGAGGGTGTCCGCCTCAGGACAGAAGTTGCGTTTTTTCGACTTGGTTGGCGATGGA

*P. falciparum*  ATAATGAGAGTATCTGCTTCTGGTCAGAAATTACGTTTCTTTGATTTGGTTGGAGATGGA

********.**.** ** **:**:*****.**.***** ** ** ********.******

*P. knowlesi*  GCCAAAATACAAGTACTAGCCAACTTCGCATTCCATGACCATACCAAGTCAAATTTCGCA

*P. falciparum*  GAGAAGATTCAAGTGTTAGCAAATTATTCTTTTCATAATCATGAGAAAGGTAATTTCGCT

*. **.**:*****. ****.** *: *:** ***.* ***.. **. :********:

*P. knowlesi*  GAAGCGTACGACAAAATAAGAAGGGGAGATATCGTAGGAATTGTGGGATTCCCAGGAAAA

*P. falciparum*  GAATGTTATGATAAGATAAGAAGAGGTGACATTGTGGGTATTGTAGGCTTTCCTGGTAAA

*** ** ** **.********.**:** ** **.**:*****.**.** **:**:***

*P. knowlesi*  AGCAAAAAGGGAGAGTTAAGTATATTCCCGAAAGAAACCATCATCCTCTCTCCCTGTCTC

*P. falciparum*  AGTAAGAAAGGTGAATTAAGTATTTTCCCTAAGGAAACTATATTACTTTCAGCTTGTTTA

** **.**.**:**.********:***** **.***** **.:*.** **: * *** *.

*P. knowlesi*  CACATGTTGCCAATGAAGTATGGATTGAAGGACACAGAAATTAGGTCACGACAGAGGTAC

*P. falciparum*  CATATGTTACCTATGAAATATGGTTTAAAAGATACTGAAATAAGATATAGACAAAGATAT

** *****.**:*****.*****:**.**.** **:*****:**.*.:.****.**.**

*P. knowlesi*  TTAGATTTAATGATAAACGAATCCACCAGAAGTACCTTTATCACCAGAACAAAAATTATT

*P. falciparum*  TTAGATTTATTAATAAATGAATCATCTCGACATACTTTTGTAACAAGAACAAAAATAATT

*********:*.***** *****.:* .**..*** ***.*.**.***********:***

*P. knowlesi*  AATTATTTAAGAAATTTTCTCAACGATAGAGGATTCATAGAAGTAGAAACCCCAACCATG

*P. falciparum*  AATTTCTTAAGAAATTTTTTAAATGAAAGAGGTTTCTTTGAAGTAGAAACACCAATGATG

****: ************ *.** **:*****:***:*:***********.**** ***

*P. knowlesi*  AATTTAGTAGCGGGGGGTGCAAATGCTAAGCCATTTATTACCCACCATAATGACTTAGAC

*P. falciparum*  AATTTAATAGCCGGTGGAGCAAATGCACGACCATTTATTACACATCATAATGATTTAGAT

******.**** ** **:********:...***********.** ******** *****

*P. knowlesi*  TTGGATCTCTACCTCCGTATTGCTACGGAGCTACCTCTAAAAATGTTAATCGTAGGAGGG

*P. falciparum*  TTAGATCTATATTTACGAATAGCTACTGAATTACCTTTAAAAATGTTAATAGTAGGTGGT

**.*****.** *.**:**:***** **. ***** *************.*****:**

*P. knowlesi*  ATAGATAAAGTCTATGAAATAGGAAAGGTGTTCAGAAATGAAGGAATAGATAATACCCAT

*P. falciparum*  ATAGATAAAGTCTATGAAATTGGTAAAGTATTTAGAAATGAAGGTATAGATAATACACAT

********************:**:**.**.** ***********:***********.***

*P. knowlesi*  AACCCTGAGTTTACGTCATGTGAATTTTATTGGGCCTATGCAGATTTTTATGATCTCATC

*P. falciparum*  AATCCTGAATTTACTTCGTGTGAATTTTATTGGGCATATGCTGATTATAATGATTTAATA

** *****.***** **.*****************.*****:****:*:***** *.**.

*P. knowlesi*  AAATGGTCAGAAGATTTTTTTTCCACTTTAGTTATGCACTTATTTGGGACTTATAAAATT

*P. falciparum*  AAATGGTCAGAAGATTTCTTCTCACAATTAGTATATCATTTATTTGGTACATATAAAATT

***************** ** **...:*****::: ** ******** **:*********

*P. knowlesi*  TTGTATAATAAAGATGGCCCAGATAAAGATCCCATAGAAATAGATTTCACCCCTCCCTAT

*P. falciparum*  TCATATAATAAAGATGGTCCAGAAAATCAACCGATAGAAATAGATTTCACACCACCTTAT

* .************** *****:**: *:** *****************.**:** ***

*P. knowlesi*  CCTAAAGTTTCCATCGTGGAGGAGTTGGAAAAATTAACCAACACAAAGTTAGAACAACCA

*P. falciparum*  CCTAAGGTTTCCATTGTAGAGGAAATAGAAAAAGTAACCAACACCATATTAGAGCAACCA

*****.******** **.*****.:*.****** **********.*:.*****.******

*P. knowlesi*  TTTGACTCTCCAGAAACAATAAACAAAATGATCAATTTGATTAAAGAAAATAAGATCGAA

*P. falciparum*  TTCGATTCAAATGAAACTATTGAAAAAATGATTAATATTATTAAAGAACATAAAATCGAA

** ** **:..:*****:**:.*.******** ***:* *********.****.******

*P. knowlesi*  ATGCCAAACCCTCCGACTGCTGCAAAACTACTAGATCAGCTGGCTTCCCACTTTATAGAA

*P. falciparum*  TTACCTAATCCTCCGACAGCTGCCAAATTATTAGATCAACTAGCTTCTCATTTTATAGAA

:*.**:** ********:*****.*** ** *******.**.***** ** *********

*P. knowlesi*  AATCAATATCCTAACAAACCTTTCTTTATTATTGAGCACCCACAAATTATGAGTCCCTTA

*P. falciparum*  AATAAATATAACGATAAACCATTTTTCATTGTTGAACATCCACAAATTATGAGCCCCCTT

***.*****.. .* *****:** ** ***.****.** ************** *** *:

*P. knowlesi*  GCAAAATATCATAGATCCAAACCAGGGTTAACTGAACGTTTGGAAATGTTTATCTGTGGG

*P. falciparum*  GCAAAATACCACAGAACCAAACCTGGGCTCACAGAAAGATTAGAAATGTTTATTTGTGGA

******** ** ***:*******:*** *.**:***.*:**.*********** *****.

*P. knowlesi*  AAGGAAGTTCTAAATGCTTACACAGAATTGAATGACCCTTTTAAACAGAAGGAATGTTTT

*P. falciparum*  AAAGAAGTTCTAAATGCTTACACAGAATTAAACGATCCTTTTAAACAAAAAGAATGTTTC

**.**************************.** ** ***********.**.********

*P. knowlesi*  TCAGCGCAGCAAAAGGATAGGGAAAAGGGAGATGCGGAGGCCTTTCAGTTTGATGCTCCA

*P. falciparum*  AAATTACAACAAAAAGATAGAGAAAAAGGTGATACTGAAGCAGCACAACTCGATTCGGCA

:.* .**.*****.*****.*****.**:***.* **.**. :**. * *** * **

*P. knowlesi*  TATTGTACATCCTTGGAATATGGACTACCACCCACAGGTGGTCTTGGCCTCGGTATAGAC

*P. falciparum*  TTCTGCACTTCTCTAGAATATGGTTTACCACCTACCGGAGGCTTAGGATTAGGTATTGAT

*: ** **:** *.********: ******* **.**:** *:**. *.*****:**

*P. knowlesi*  CGAATCACTATGTTCCTAACCAACAAGAATTGTATTAAGGATGTGATCCTCTTTCCAACC

*P. falciparum*  AGAATTACTATGTTTTTAACCAACAAGAATTCCATAAAAGATGTTATATTATTTCCCACT

.**** ******** *************** **:**.***** **. *.*****.**

*P. knowlesi*  ATGCGCCCTGCGAATTAA

*P. falciparum*  ATGCGACCAGCAAATTGA

*****.**:**.****.*

Supplementary Figure S4: Alignment of *P. knowlesi* and *P. falciparum* KRS genomic sequences – Primer annealing sites are highlighted in yellow, region included in the mutation library is highlighted in green with 5’ and 3’ regions of sequence which are also included as part of the library construction are in orange.


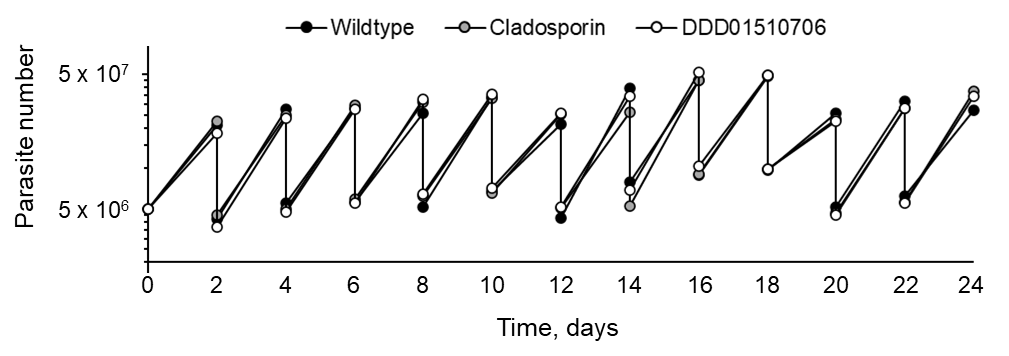


Supplementary Figure S5: Growth curve of mutation library during drug selection**.**


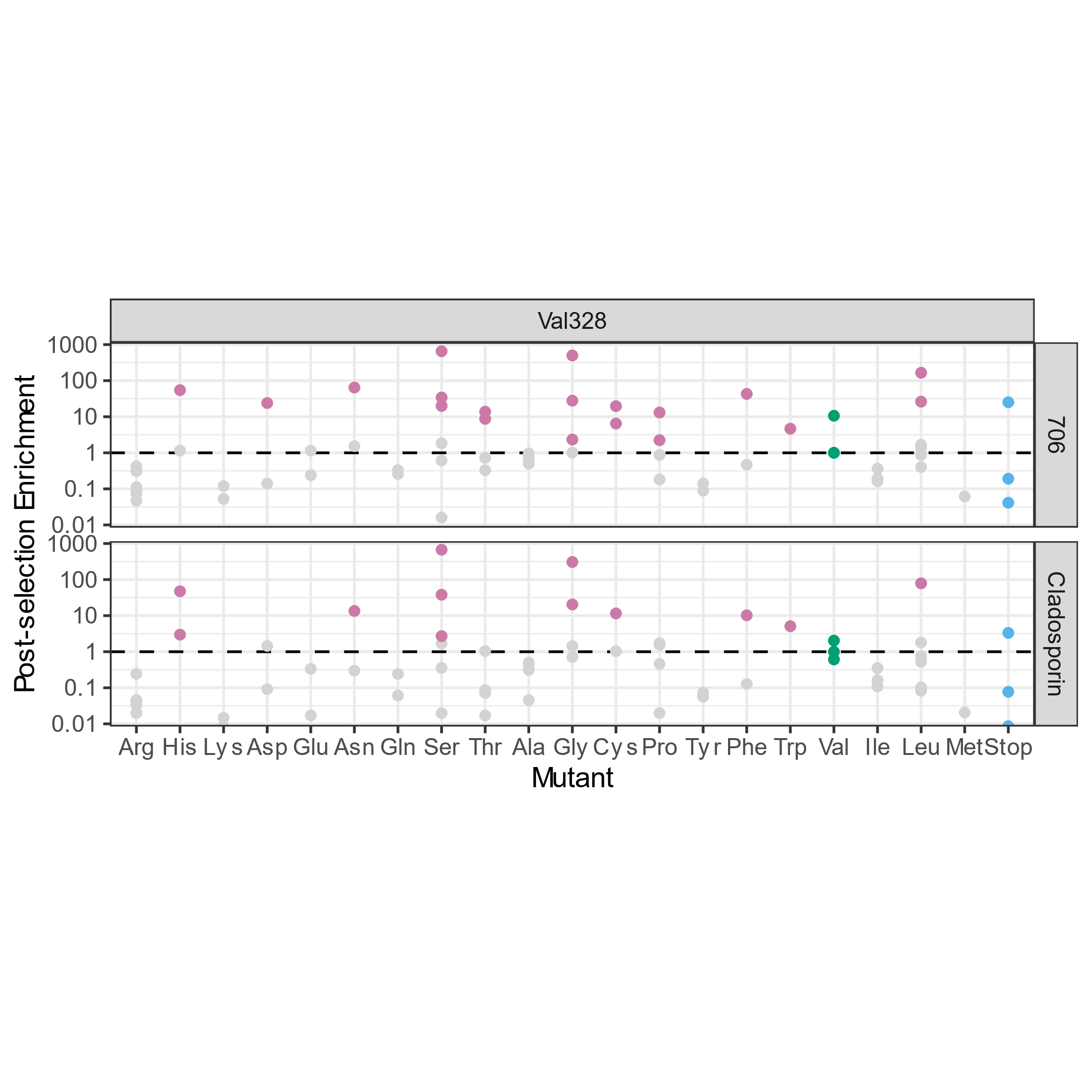


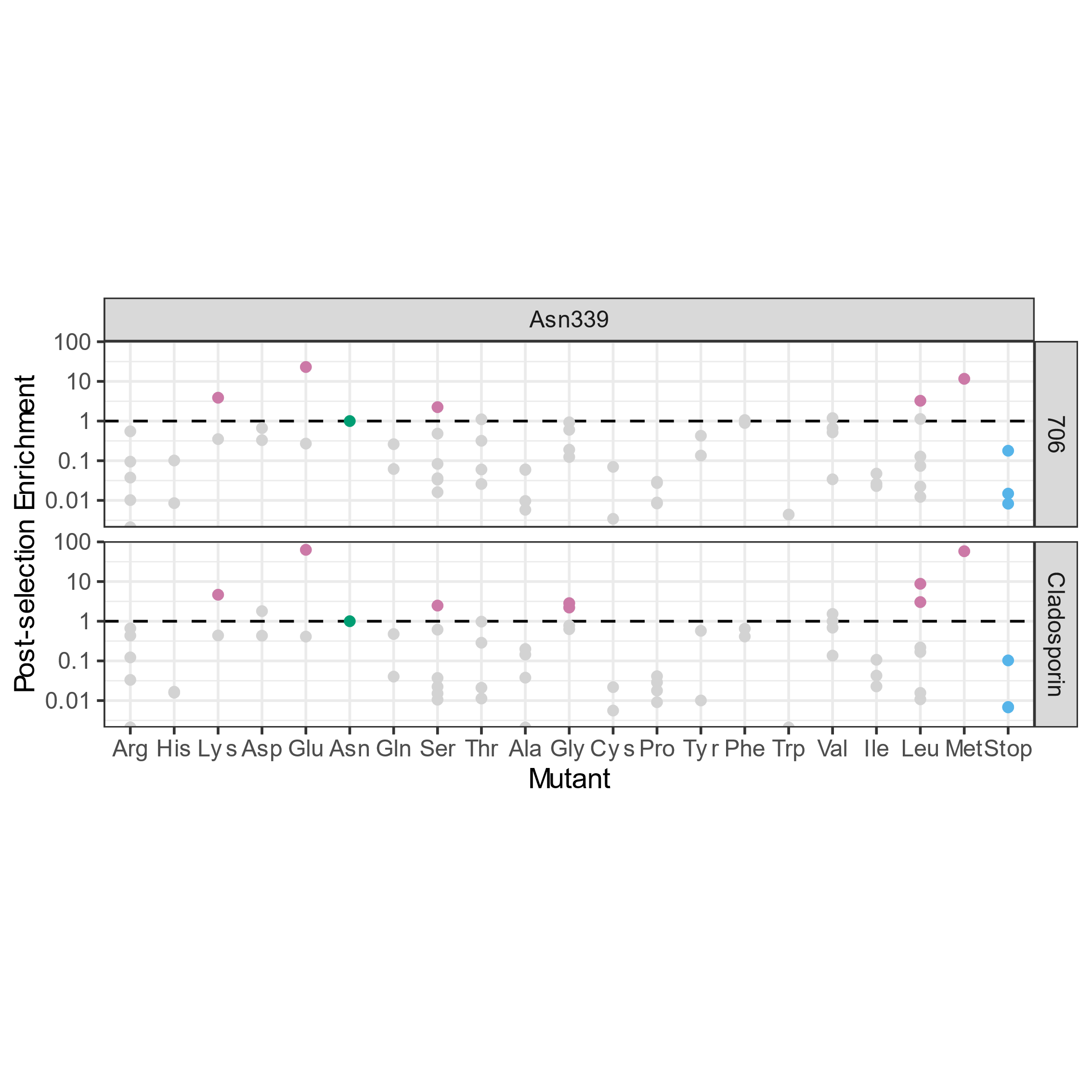


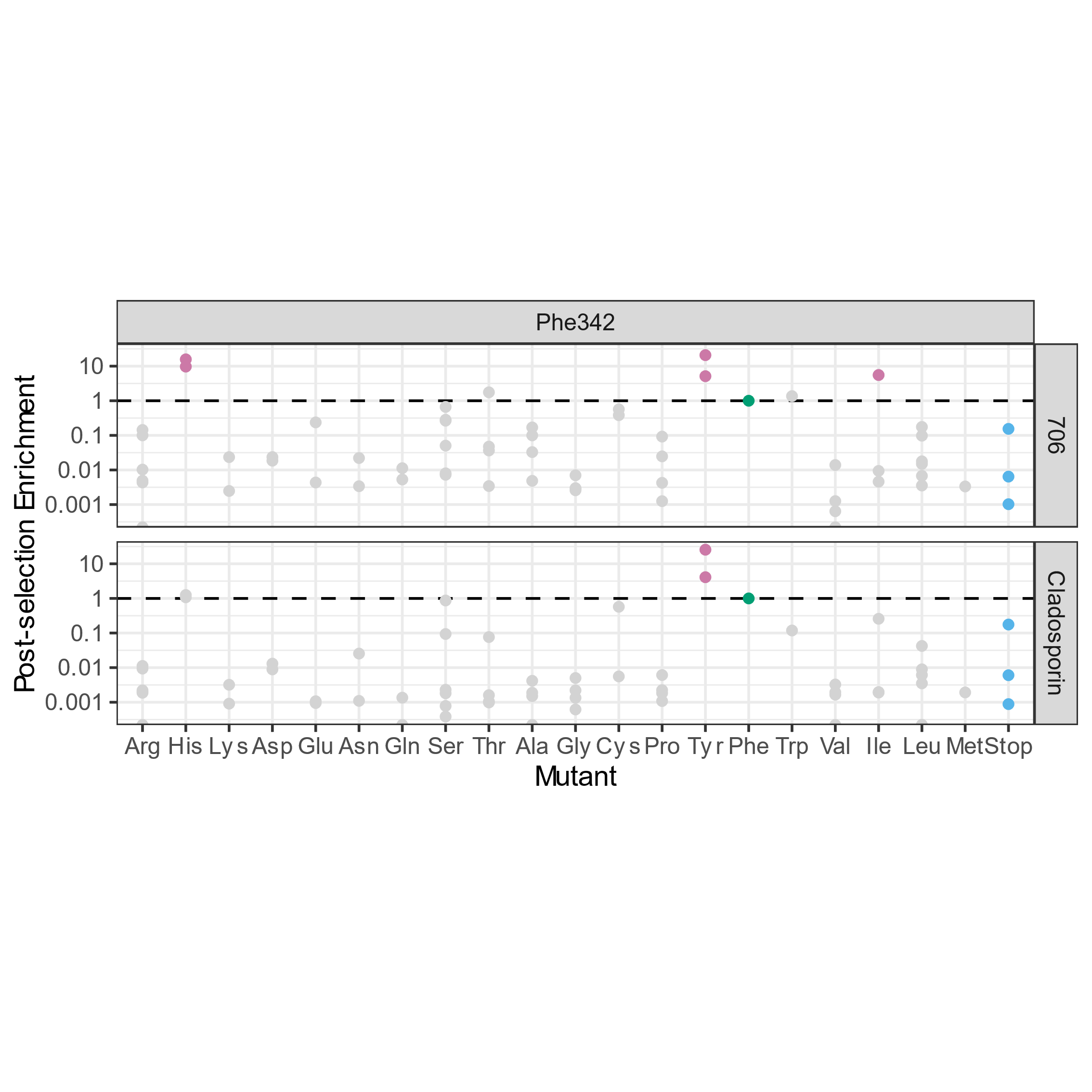


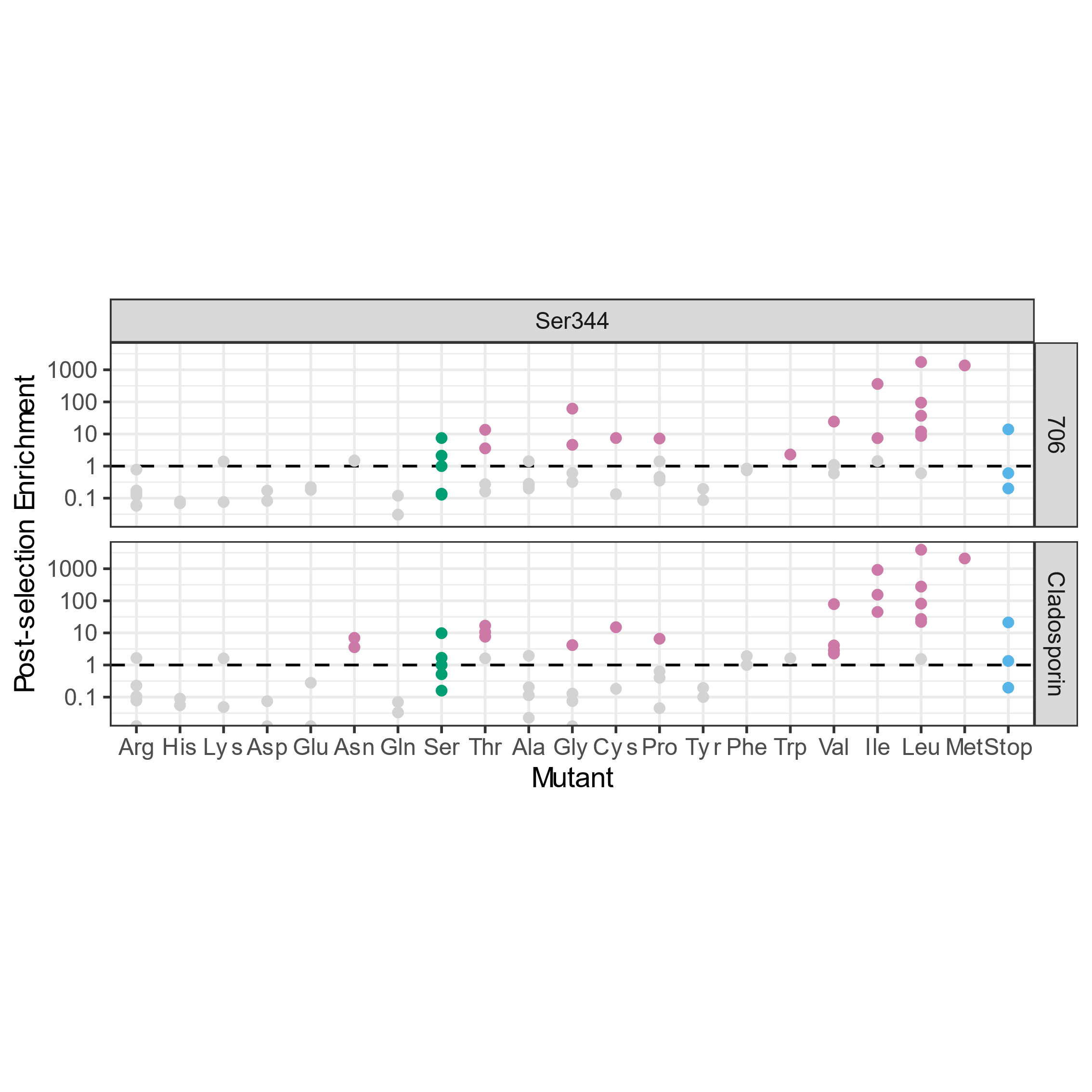


Supplementary Figure S6: Specific nucleotide sequence enrichment at positions V328, N339, F342 and S344 following selection with KRS inhibitors **-** Highlighted mutation types: synonymous (green), terminating (blue) and highly significant resistance mutants (pink). The enrichment score has been additionally normalised to the mean of the synonymous changes for clarity.

**
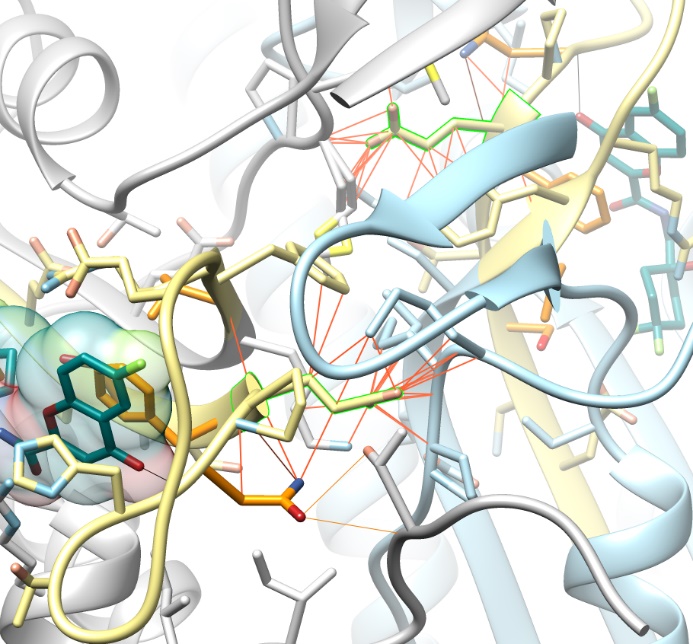
**

Supplementary Figure S7: Structural context of N339 in KRS-DDD01510706 complex (PDB ID: 6HCU)**.** N339 is oriented towards the homomeric interface. It is involved in numerous non-bonded interactions and is H-bonded to T578. The disruption of these interactions could potentially affect loop dynamics with consequences for drug binding.


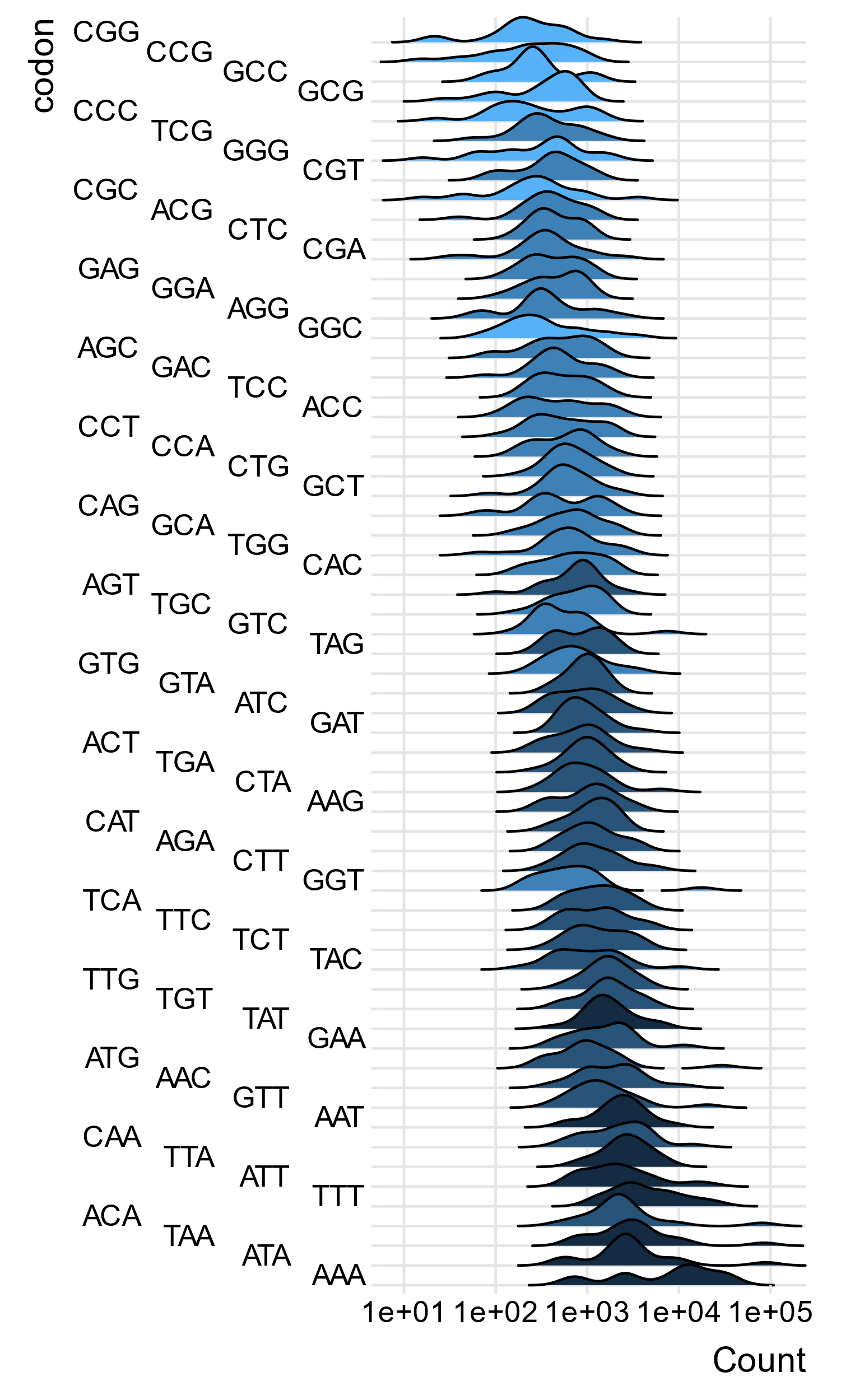


## **Supplementary Figure S8**: Nucleotide ratio content of synthesised degenerate oligonucleotides

**
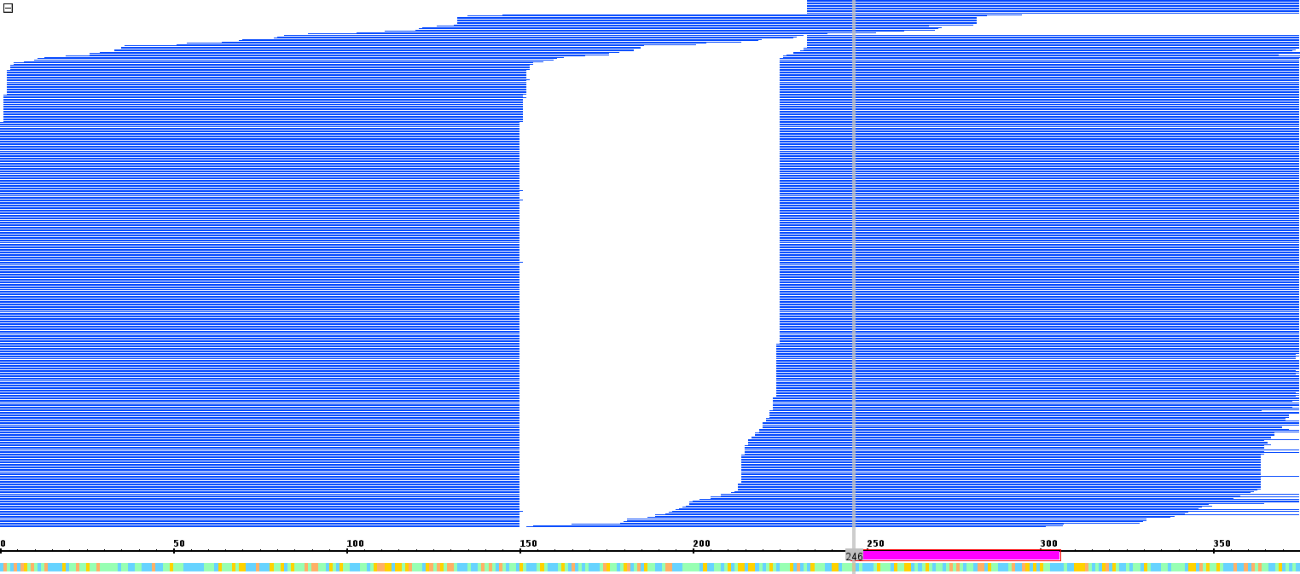
**

Supplementary Figure S9: Alignment of sequencing reads showing the mutation library region. Library region shown in magenta. Figure created with IntegratedGenomeBrowser (1).


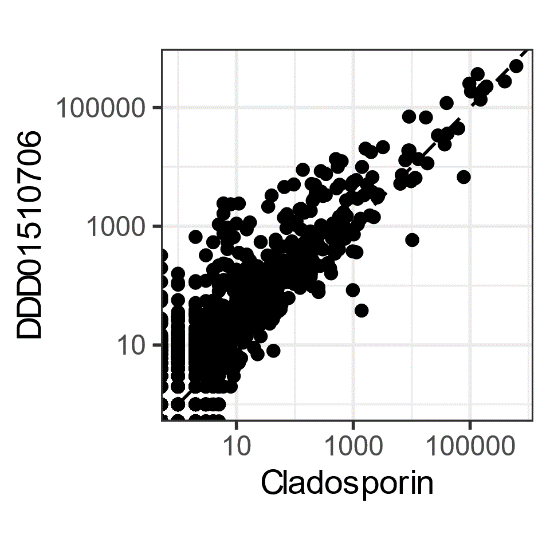


Supplementary Figure S10: Correlation between post-selection read counts following treatment with both compounds**.**

# **Supplementary Tables**

## **Supplementary Table S1**: Oligonucleotides used in this study.

| **Name** | **Sequence (5´ - 3´)** |
| --- | --- |
| PkKRSOE_f | cgtacccgggATGTTCAGATATTTACTCCCCATCC |
| PkKRSOE_r | cgtaccgcggTTAATTCGCAGGGCGCATGGTT |
| PfKRSmutlibPCR_f | TCATCTCGACATACTTTTGTAACAAG |
| PfKRSmutlibPCR_r | ATATACTAATTGTGAGAAGAAATCTTCTG |
| PfKRSmutWTA | GTCTATGAAATTGGTAAAGTATTTAGAAATGAAGGTATAGATAATACACATAATCCTGAATTTACTTCGTGTGAATTTTATTGGGCATATGCT |
| PfKRSmutWTB | ATCAGCATATGCCCAATAAAATTCACACGAAGTAAATTCAGGATTATGTGTATTATCTATACCTTCATTTCTAAATACTTTACCAATTTCATA |
| PfKRSmut1A | GTCTATGAAATTGGTAAANNNTTTAGAAATGAAGGTATAGATAATACACATAATCCTGAATTTACTTCGTGTGAATTTTATTGGGCATATGCT |
| PfKRSmut2A | GTCTATGAAATTGGTAAAGTANNNAGAAATGAAGGTATAGATAATACACATAATCCTGAATTTACTTCGTGTGAATTTTATTGGGCATATGCT |
| PfKRSmut3A | GTCTATGAAATTGGTAAAGTATTTNNNAATGAAGGTATAGATAATACACATAATCCTGAATTTACTTCGTGTGAATTTTATTGGGCATATGCT |
| PfKRSmut4A | GTCTATGAAATTGGTAAAGTATTTAGANNNGAAGGTATAGATAATACACATAATCCTGAATTTACTTCGTGTGAATTTTATTGGGCATATGCT |
| PfKRSmut5A | GTCTATGAAATTGGTAAAGTATTTAGAAATNNNGGTATAGATAATACACATAATCCTGAATTTACTTCGTGTGAATTTTATTGGGCATATGCT |
| PfKRSmut6A | GTCTATGAAATTGGTAAAGTATTTAGAAATGAANNNATAGATAATACACATAATCCTGAATTTACTTCGTGTGAATTTTATTGGGCATATGCT |
| PfKRSmut7A | GTCTATGAAATTGGTAAAGTATTTAGAAATGAAGGTNNNGATAATACACATAATCCTGAATTTACTTCGTGTGAATTTTATTGGGCATATGCT |
| PfKRSmut8A | GTCTATGAAATTGGTAAAGTATTTAGAAATGAAGGTATANNNAATACACATAATCCTGAATTTACTTCGTGTGAATTTTATTGGGCATATGCT |
| PfKRSmut9A | GTCTATGAAATTGGTAAAGTATTTAGAAATGAAGGTATAGATNNNACACATAATCCTGAATTTACTTCGTGTGAATTTTATTGGGCATATGCT |
| PfKRSmut10A | GTCTATGAAATTGGTAAAGTATTTAGAAATGAAGGTATAGATAATNNNCATAATCCTGAATTTACTTCGTGTGAATTTTATTGGGCATATGCT |
| PfKRSmut11A | GTCTATGAAATTGGTAAAGTATTTAGAAATGAAGGTATAGATAATACANNNAATCCTGAATTTACTTCGTGTGAATTTTATTGGGCATATGCT |
| PfKRSmut12A | GTCTATGAAATTGGTAAAGTATTTAGAAATGAAGGTATAGATAATACACATNNNCCTGAATTTACTTCGTGTGAATTTTATTGGGCATATGCT |
| PfKRSmut13A | GTCTATGAAATTGGTAAAGTATTTAGAAATGAAGGTATAGATAATACACATAATNNNGAATTTACTTCGTGTGAATTTTATTGGGCATATGCT |
| PfKRSmut14A | GTCTATGAAATTGGTAAAGTATTTAGAAATGAAGGTATAGATAATACACATAATCCTNNNTTTACTTCGTGTGAATTTTATTGGGCATATGCT |
| PfKRSmut15A | GTCTATGAAATTGGTAAAGTATTTAGAAATGAAGGTATAGATAATACACATAATCCTGAANNNACTTCGTGTGAATTTTATTGGGCATATGCT |
| PfKRSmut16A | GTCTATGAAATTGGTAAAGTATTTAGAAATGAAGGTATAGATAATACACATAATCCTGAATTTNNNTCGTGTGAATTTTATTGGGCATATGCT |
| PfKRSmut17A | GTCTATGAAATTGGTAAAGTATTTAGAAATGAAGGTATAGATAATACACATAATCCTGAATTTACTNNNTGTGAATTTTATTGGGCATATGCT |
| PfKRSmut18A | GTCTATGAAATTGGTAAAGTATTTAGAAATGAAGGTATAGATAATACACATAATCCTGAATTTACTTCGNNNGAATTTTATTGGGCATATGCT |
| PfKRSmut19A | GTCTATGAAATTGGTAAAGTATTTAGAAATGAAGGTATAGATAATACACATAATCCTGAATTTACTTCGTGTNNNTTTTATTGGGCATATGCT |
| PfKRSmut20A | GTCTATGAAATTGGTAAAGTATTTAGAAATGAAGGTATAGATAATACACATAATCCTGAATTTACTTCGTGTGAANNNTATTGGGCATATGCT |
| PfKRSmut20B | ATCAGCATATGCCCAATANNNTTCACACGAAGTAAATTCAGGATTATGTGTATTATCTATACCTTCATTTCTAAATACTTTACCAATTTCATA |
| PfKRSmut19B | ATCAGCATATGCCCAATAAAANNNACACGAAGTAAATTCAGGATTATGTGTATTATCTATACCTTCATTTCTAAATACTTTACCAATTTCATA |
| PfKRSmut18B | ATCAGCATATGCCCAATAAAATTCNNNCGAAGTAAATTCAGGATTATGTGTATTATCTATACCTTCATTTCTAAATACTTTACCAATTTCATA |
| PfKRSmut17B | ATCAGCATATGCCCAATAAAATTCACANNNAGTAAATTCAGGATTATGTGTATTATCTATACCTTCATTTCTAAATACTTTACCAATTTCATA |
| PfKRSmut16B | ATCAGCATATGCCCAATAAAATTCACACGANNNAAATTCAGGATTATGTGTATTATCTATACCTTCATTTCTAAATACTTTACCAATTTCATA |
| PfKRSmut15B | ATCAGCATATGCCCAATAAAATTCACACGAAGTNNNTTCAGGATTATGTGTATTATCTATACCTTCATTTCTAAATACTTTACCAATTTCATA |
| PfKRSmut14B | ATCAGCATATGCCCAATAAAATTCACACGAAGTAAANNNAGGATTATGTGTATTATCTATACCTTCATTTCTAAATACTTTACCAATTTCATA |
| PfKRSmut13B | ATCAGCATATGCCCAATAAAATTCACACGAAGTAAATTCNNNATTATGTGTATTATCTATACCTTCATTTCTAAATACTTTACCAATTTCATA |
| PfKRSmut12B | ATCAGCATATGCCCAATAAAATTCACACGAAGTAAATTCAGGNNNATGTGTATTATCTATACCTTCATTTCTAAATACTTTACCAATTTCATA |
| PfKRSmut11B | ATCAGCATATGCCCAATAAAATTCACACGAAGTAAATTCAGGATTNNNTGTATTATCTATACCTTCATTTCTAAATACTTTACCAATTTCATA |
| PfKRSmut10B | ATCAGCATATGCCCAATAAAATTCACACGAAGTAAATTCAGGATTATGNNNATTATCTATACCTTCATTTCTAAATACTTTACCAATTTCATA |
| PfKRSmut9B | ATCAGCATATGCCCAATAAAATTCACACGAAGTAAATTCAGGATTATGTGTNNNATCTATACCTTCATTTCTAAATACTTTACCAATTTCATA |
| PfKRSmut8B | ATCAGCATATGCCCAATAAAATTCACACGAAGTAAATTCAGGATTATGTGTATTNNNTATACCTTCATTTCTAAATACTTTACCAATTTCATA |
| PfKRSmut7B | ATCAGCATATGCCCAATAAAATTCACACGAAGTAAATTCAGGATTATGTGTATTATCNNNACCTTCATTTCTAAATACTTTACCAATTTCATA |
| PfKRSmut6B | ATCAGCATATGCCCAATAAAATTCACACGAAGTAAATTCAGGATTATGTGTATTATCTATNNNTTCATTTCTAAATACTTTACCAATTTCATA |
| PfKRSmut5B | ATCAGCATATGCCCAATAAAATTCACACGAAGTAAATTCAGGATTATGTGTATTATCTATACCNNNATTTCTAAATACTTTACCAATTTCATA |
| PfKRSmut4B | ATCAGCATATGCCCAATAAAATTCACACGAAGTAAATTCAGGATTATGTGTATTATCTATACCTTCNNNTCTAAATACTTTACCAATTTCATA |
| PfKRSmut3B | ATCAGCATATGCCCAATAAAATTCACACGAAGTAAATTCAGGATTATGTGTATTATCTATACCTTCATTNNNAAATACTTTACCAATTTCATA |
| PfKRSmut2B | ATCAGCATATGCCCAATAAAATTCACACGAAGTAAATTCAGGATTATGTGTATTATCTATACCTTCATTTCTNNNTACTTTACCAATTTCATA |
| PfKRSmut1B | ATCAGCATATGCCCAATAAAATTCACACGAAGTAAATTCAGGATTATGTGTATTATCTATACCTTCATTTCTAAANNNTTTACCAATTTCATA |

Supplementary Table S2: Read counts and library ratio for the pre-selected mutation library**.**

Supplementary Table S3: Read counts and library ratio following treatment with DDD01510706 normalised against pre-selected library**.**

Supplementary Table S4: Read counts and library ratio following treatment with cladosporin normalised against pre-selected library**.**

Supplementary Table S5: Selection of mutations enriched by ≥2-fold over unselected lines following selection with cladosporin or DDD01510706**.** ^1^Mutations validated in this study, ^2^mutations >5-fold enriched in DDD01510706 screen compared to the cladosporin screen. Number of changes required to generate mutation from WT is shown.

| **Mutation** | **DDD01510706 enrichment** | **Cladosporin enrichment** | **logFC (edgeR)** | **p-adj** | **Nucleotide edits** |
| --- | --- | --- | --- | --- | --- |
| S344M | 67.8 | 72.9 | 11.8 | 4.7E-06 | 2 |
| N339M | 28.5 | 55.8 | 11.2 | 5.6E-05 | 2 |
| **S344L**^1^ | **15.6** | **25.1** | **11.1** | **2.4E-05** | **1** |
| **N339E**^1^ | **28.6** | **30.5** | **11.0** | **8.6E-06** | **2** |
| **F342Y**^1^ | **12.1** | **20.5** | **10.4** | **6.1E-05** | **1** |
| T343* | 8.3 | 11.7 | 9.9 | 6.1E-05 | 3 |
| H338* | 32.4 | 15.8 | 9.8 | 8.6E-06 | 2 |
| V328G | 17.3 | 7.4 | 9.8 | 9.9E-06 | 1 |
| **V328S**^1^ | **15.4** | **10.7** | **9.6** | **1.7E-05** | **2** |
| C345D | 2.4 | 5.2 | 8.3 | 0.001 | 2 |
| N339K | 5.2 | 2.4 | 8.2 | 5.3E-05 | 1 |
| S344I | 6.1 | 13 | 8.0 | 0.003 | 3 |
| F342H^2^ | 11.8 | < 2.0 | 7.8 | 6.4E-04 | 2 |
| C345R | 2.3 | 5 | 7.3 | 0.003 | 1 |
| F329P | 5.8 | 2.4 | 7.3 | 3.0E-04 | 2 |
| V328L | 4.3 | < 2.0 | 7.3 | 3.2E-04 | 1 |
| V328H | 3.6 | 2.3 | 7.1 | 4.2E-04 | 3 |
| F329* | 2.3 | < 2.0 | 7.0 | 5.2E-04 | 2 |
| V328N | 4.3 | < 2.0 | 6.6 | 0.003 | 3 |
| V328F | 2.8 | < 2.0 | 6.6 | 0.002 | 2 |
| N339N | 2.5 | < 2.0 | 6.1 | 0.002 | 1 |
| N339F | 2.4 | < 2.0 | 5.7 | 0.004 | 2 |
| P340F | 2.5 | < 2.0 | 5.2 | 0.005 | 2 |
| T343Y | 2.2 | < 2.0 | 5.0 | 0.062 | 2 |
| H338Q | 2.4 | < 2.0 | 3.2 | 0.025 | 1 |

Supplementary Table S6: Genetic diversity of *Pf*KRS from clinical isolates. Data isolated from the PF7 database (2), green highlight indicates changes located in the compound binding site (within 7Å distance).

**References**

1. Freese, N.H., Norris, D.C. and Loraine, A.E. (2016) Integrated genome browser: visual analytics platform for genomics. *Bioinformatics*, **32**, 2089-2095.

2. Abdel Hamid, M.M., Abdelraheem, M.H., Acheampong, D.O., Ahouidi, A., Ali, M., Almagro-Garcia, J., Amambua-Ngwa, A., Amaratunga, C., Amenga-Etego, L., Andagalu, B. *et al.* (2023) Pf7: an open dataset of Plasmodium falciparum genome variation in 20,000 worldwide samples. *Wellcome Open Res*, **8**, 22.
